# Short-Term High-Fat Diet Consumption Reduces Hypothalamic Expression of the Nicotinic Acetylcholine Receptor α7 Subunit (α7nAChR) and Affects the Anti-inflammatory Response in a Mouse Model of Sepsis

**DOI:** 10.3389/fimmu.2019.00565

**Published:** 2019-03-22

**Authors:** Anelise Cristina Parras Souza, Camilla Mendes Souza, Camila Libardi Amaral, Simone Ferreira Lemes, Leticia Foglia Santucci, Marciane Milanski, Adriana Souza Torsoni, Marcio Alberto Torsoni

**Affiliations:** ^1^School of Applied Sciences, University of Campinas, Limeira, Brazil; ^2^Obesity and Comorbidities Research Center, State University of Campinas, Limeira, Brazil

**Keywords:** α7nAChR, high-fat diet, short-term, LPS, sepsis, inflammation

## Abstract

Sepsis is one of the leading causes of death in hospitalized patients and the chronic and low-grade inflammation observed in obesity seems to worsen susceptibility and morbidity of infections. However, little is known with respect to a short-term high-fat diet (HFD) and its role in the development of sepsis. Here, we show for the first time, that short-term HFD consumption impairs early nicotinic acetylcholine receptor α7 subunit (α7nAChR)- mediated signaling, one of the major components of the cholinergic anti-inflammatory pathway, with a focus on hypothalamic inflammation and innate immune response. Mice were randomized to a HFD or standard chow (SC) for 3 days, and sepsis was subsequently induced by a lethal intraperitoneal (i.p.) injection of lipopolysaccharide (LPS) or by cecal ligation and puncture (CLP) surgery. In a separate experiment, both groups received LPS (i.p.) or LPS (i.p.) in conjunction with the selective α7nAChR agonist, PNU-282987 (i.p. or intracerebroventricular; i.c.v.), and were sacrificed 2 h after the challenge. Short-term HFD consumption significantly reduced the α7nAChR mRNA and protein levels in the hypothalamus and liver (*p* < 0.05). Immunofluorescence microscopy demonstrated lower cholinergic receptor nicotinic α7 subunit (α7nAChR)+ cells in the arcuate nucleus (ARC) (α7nAChR+ cells in SC = 216 and HFD = 84) and increased F4/80+ cells in the ARC (2.6-fold) and median eminence (ME) (1.6-fold), which can contribute to neuronal damage. Glial fibrillary acidic protein (GFAP)+ cells and neuronal nuclear antigen (NeuN)+ cells were also increased following consumption of HFD. The HFD-fed mice died quickly after a lethal dose of LPS or following CLP surgery (2-fold compared with SC). The LPS challenge raised most cytokine levels in both groups; however, higher levels of TNF-α (Spleen and liver), IL-1β and IL-6 (in all tissues evaluated) were observed in HFD-fed mice. Moreover, PNU-282987 administration (i.p. or i.c.v.) reduced the levels of inflammatory markers in the hypothalamus following LPS injection. Nevertheless, when the i.c.v. injection of PNU-282987 was performed the anti-inflammatory effect was much smaller in HFD-fed mice than SC-fed mice. Here, we provide evidence that a short-term HFD impairs early α7nAChR expression in central and peripheral tissues, contributing to a higher probability of death in sepsis.

## Introduction

The consumption of a high-fat diet (HFD) is well-known to be associated with a chronic low-grade inflammatory process. Dietary saturated fatty acids can trigger central and peripheral inflammation upon binding and activation of the Toll-like receptor 4 (TLR4), a major component of the innate immune system (1, 2). HFD-induced inflammation is mainly characterized by adipocyte secretion of pro-inflammatory cytokines, infiltration of activated immune cells, and elevation of plasma levels of gut-derived lipopolysaccharide (LPS); a state known as metabolic endotoxemia. This abnormal secretion of pro-inflammatory cytokines leads to the impairment of insulin signaling in central and peripheral tissues, and subsequently, to the development of several other obesity-related comorbidities (3).

Studies have shown that, unlike in peripheral tissues, only a very short-term exposure to a HFD promotes inflammation in the hypothalamus (4, 5). Thaler et al. (4) observed an increase in inflammatory markers in the hypothalamus of rodents fed a HFD for only 1 day. Within 3 days of HFD consumption, an accumulation of microglia and astrocytes was found in the hypothalamic arcuate nucleus (ARC), an indicator of reactive gliosis. More recently, Waise et al. (5) showed adipose neutrophil infiltration and recruitment of M1 macrophages in the nodose ganglion in mice following 1 day of a HFD. These studies suggest that HFD-induced hypothalamic inflammation occurs prior to a systemic inflammatory response; and thus, may represent a potential target to prevent the subsequent inflammation of peripheral tissues.

Several anti-inflammatory pathways take part in the control of HFD-induced inflammation, including the cholinergic pathway (6). This neuronal mechanism attenuates the inflammatory response and limits cytokine release through the activation of the nicotinic acetylcholine receptor α7 subunit (α7nAChR) (7, 8), which is widely expressed in brain cells and cytokine-producing immune cells such as macrophages, dendritic cells, and T cells (9). Central activation of the cholinergic pathway has been shown to reduce manipulation-induced intestinal inflammation and prevent postoperative ileus in a manner dependent on the vagus nerve (10, 11). Previous studies have examined the connection between activation of the cholinergic anti-inflammatory pathway and HFD-induced inflammation (12, 13). However, these studies involved the consumption of a HFD over an extended period to reflect obesity of an adult individual; and thus, may not correspond to the inflammatory modifications that occur in childhood obesity or in the initial stages of adult obesity.

Here we hypothesize that short-term HFD consumption impairs α7nAChR expression in central and peripheral tissues contributing to exacerbate inflammatory response making these mice more susceptible to sepsis development after an immune challenge. To the best of our knowledge, this is the first study to show the effect of short-term HFD consumption on α7nAChR expression and the outcome of an inflammatory response and sepsis.

## Methods

### Animals

The experimental procedures involving mice were performed in accordance with the guidelines of the Brazilian Society of Science in Laboratory Animals (SBCAL) and were approved by the Ethical Committee for Animal Use (ECAU) (ID protocol 41841) of the University of Campinas (UNICAMP). *Swiss* male mice (8-weeks old, weight 30–40 g) utilized for the present study were obtained from the university breeding colony. Mice were housed in groups of 5 individuals in a room with controlled temperature (22–24°C) and a 12-h light/dark cycle, with access to water and food *ad libitum*. All efforts were made to minimize the number of animals used.

### Experimental Design

In the present study, all mice were randomly divided into two groups: one group was fed standard chow (SC) (NuvilabR CR-1, Nuvital, PR – Brazil) and the other group was fed a high-fat diet (HFD; 60%) for 3 days. The HFD was prepared in our laboratory according to the AIN-93G modified for high-fat (60%) content.

#### Inflammatory Response

To evaluate the inflammatory response to sepsis induction, mice were separated as shown below:

##### Experiment 1

After 3 days of SC or a HFD, mice were injected intraperitoneally (i.p.) with lipopolysaccharide (LPS) at 12 mg/kg (*Escherichia coli* serotype O111:B4. L2630, Sigma-Aldrich, St. Louis MO, USA) or saline vehicle (0.9%). Mice were sacrificed 2 h after the challenge and the tissues (hypothalamus, spleen, and liver) were collected.

After 3 days of SC or a HFD, a further mice were injected i.p. with LPS at 12 mg/kg in conjunction with PNU-282987 (Sigma-Aldrich, MO, USA) at 3 mg/kg or DMSO vehicle. PNU-282987 was administered 80 min after LPS injection. Mice were sacrificed and the tissues (hypothalamus, spleen, and liver) were collected for analysis 40 min after PNU-282987 administration.

In a separate study, the inflammatory response was assessed in mice fed SC or a HFD following cecal ligation and puncture (CLP) surgery, which is considered the gold standard for the induction of sepsis. Mice were sacrificed 24 h after the surgery, and the hypothalamus was collected for molecular analyzes.

##### Experiment 2

To explore the underlying mechanisms of central α7nAChR-mediated anti-inflammatory effects in mice after being fed SC or a HFD, PNU-282987 was administered by intracerebroventricular (i.c.v.) injection (2 μL 5 × 10^−6^ M solution) 80 min after LPS challenge, and the mice were euthanized after a further 40 min.

##### Survival rate

To determine the survival rate, both groups (SC and HFD) were weighed and a lethal dose of LPS at 30 mg/kg was administered i.p. as previously described (14). Simultaneously, sepsis was induced in mice fed SC or a HFD by CLP surgery. The survival rate was recorded every 2 h.

### Tissue Extraction

All mice were anesthetized (200 mg/kg BW ketamine and 5 mg/kg BW diazepam, i.p.) and subsequently euthanized for the extraction of the hypothalamus, liver, and spleen. The extracted tissues were snap-frozen on dry ice for storage at −80°C until processing for qRT-PCR or western blotting analysis.

### Cecal Ligation and Puncture (CLP) Surgery

Mice fed SC or a HFD for 3 days were separated into four groups: SC-sham and HFD-sham (mice that received only the laparotomy incision), and SC-CLP and HFD-CLP (mice in which sepsis was induced by CLP).

Mice received inhalation anesthesia and were subjected to a longitudinal incision in the abdomen, such that their cecum was exposed outside the peritoneal cavity, and ligation was performed with a silk line for suturing. The exposed cecum was punctured with a needle and the fecal content was extravasated into the peritoneal cavity. Subsequently, the cecum was reinserted into the original position and the abdomen was sutured.

As a control for the surgical variable, the other half of the animals (HFD-Sham and SC-Sham) were subjected only to the laparotomy incision in the abdomen for exposure of the cecum; however, there was no puncture or extravasation of the fecal contents.

Following the procedure, the animals were fed a standard control diet for 24 h, and subsequently sacrificed for collection of the hypothalamus.

### Stereotaxic Surgery and Intracerebroventricular (i.c.v.) Cannulation

Mice fed SC or a HFD received an i.p. injection of anesthesia as previously described (15), and were placed in a stereotaxic instrument. Afterward, a 26-gauge needle of a 10-μl Hamilton syringe was inserted into the lateral ventricle through a cranial burr hole at the following coordinates relative to the bregma: anterior/posterior axis, 0.34 mm from the bregma to the rear; lateral, 1 mm from the midline; dorsoventral, 2.2 mm from the surface of the skull. Dental acrylic glue was added to secure the cannula following correct positioning. After surgery, the animals were allowed to recover from anesthesia on a warm pad. The cannula placement was tested by measuring the dipsogenic response to an i.c.v. injection of angiotensin II (2 μl 10^−6^ M solution) (Sigma-Aldrich, MO, USA). The time and dose of PNU-282987 treatment were standardized from time-course and dose-response experiments (data not shown).

### Real-Time PCR Analysis

The total RNA was extracted from the hypothalamus, liver, and spleen using Trizol^®^ Reagent (Invitrogen Corporation, CA, USA) according to the manufacturer's recommendations, and quantitated using a Nanodrop ND-2000 (Thermo Electron, WI, USA). Reverse transcription was performed with 3 μg total RNA and a High Capacity cDNA Reverse Transcription kit (Life Technologies Corporation, Carlsbad, CA, USA). Relative expression was determined using the TaqMan™ detection system and primers for the target genes: Mm01312230_m1 for CHRNA7; Mm00443258_m1 for TNF-α; Mm00434228_m1 for IL-1β; Mm00446190_m1 for IL-6; Mm01288386_m1 for IL-10; Mm00657889_mH for Chil3; Mm00436450_m1 for CXCL2; Mm00441242_m1 for CCL2; and Mm00436454_m1 for CX3CL1 (Life Technologies Corporation, Carlsbad, CA, USA). GAPDH was used as the endogenous control (4352339E mouse GAPDH, Life Technologies Corporation, Carlsbad, CA, USA). Each PCR reaction contained 20 ng cDNA. Gene expression was quantitated by real-time PCR performed on an ABI Prism 7500 Fast platform. Data were analyzed using a Sequence Detection System 2.0.5 (Life Technologies Corporation, Carlsbad, CA, USA), and expressed as relative values determined by the comparative threshold cycle (Ct) method (2–ΔΔ Ct), according to the manufacturer's recommendations (16).

### Western Blotting

Fragments of tissues (hypothalamus, spleen, and liver) were homogenized separately in freshly prepared ice-cold buffer [10 mM EDTA; 100 mM Tris, pH 7.4; 100 mM sodium pyrophosphate; 10 mM sodium fluoride; 10 mM sodium vanadate; 2 mM PMSF; 0.01 mg/mL aprotinin; 1% (v/v) Triton X-100]. The insoluble material was removed by centrifugation (11,500 rpm) for 30 min at 4°C. The protein concentration of the supernatant was determined by the Bradford dye-binding method (1976). The supernatant was diluted in Laemmli sample buffer and boiled for 5 min prior to separation by SDS-PAGE using a miniature slab gel apparatus (Bio Rad, Richmond, CA, USA). Electrotransfer of the proteins from the gel to a nitrocellulose membrane was performed for 90 min at 120 V (constant). The membranes were subsequently blocked in 5% dried milk, washed three times in PBS, and incubated with specific primary antibodies overnight at 4°C. Following incubation, the membranes were washed a further three times in PBST and incubated with HRP-conjugated secondary antibodies (KPL, Gaithersburg, MD, USA) for 1 h at room temperature. Proteins recognized by the secondary antibodies were detected using SuperSignal™ West Pico Chemiluminescent Substrate (Thermo Scientific, Waltham, MA, USA) and a Chemi Gel documentation (New England Biogroup. Atkinson, WI, USA). Band intensities were quantitated by optical densitometry using the Scion Image Software (ScionCorp, MD, USA), and normalized to GAPDH (16, 17).

### Immunofluorescence and Confocal Microscopy

Mice in both groups (SC and HFD) were perfused with 4% PFA. Brains were extracted and fixed in 4% PFA, and subsequently embedded in Tissue-Tek (Sakura, Torrance, CA, USA), frozen, and cut into 15-μm thick coronal sections. Slides were incubated in blocking solution (1% bovine albumin; Sigma-Aldrich, St. Louis, MO, USA) for 90 min, followed by incubation with specific primary antibodies overnight at 4°C. The primary antibodies used were: anti-CHRNA7 1:500 (bs-1049r—Bioss, Woburn, MA, USA); anti-GFAP 1:250 (sc-33673—Santa Cruz Biotechnology, Santa Cruz, CA, USA); anti-F4/80 1:500 (ab6640—Abcam, Cambridge, MA, USA), and anti-NeuN (ab-104224—Abcam, Cambridge, MA, USA). Slides were washed and incubated with appropriate secondary antibodies for 90 min. The secondary antibodies used were: Alexa 488-conjugated donkey anti-rabbit 1:500 (ab21208, Abcam, Cambridge, MA, USA) and Alexa 594-conjugated goat anti-mouse 1:500 (ab11032, Abcam, Cambridge, MA, USA). TO-PRO-3^®^ Iodide was used for nuclear labeling (1:1,000; Life Technologies, Carlsbad, CA, USA). Slides were visualized and images captured using a TCS SP5II Leica confocal microscope (Leica Microsystems, Wetzlar, Hesse, Germany). The numbers of CHRNA7+, GFAP+, F4/80+, and NeuN+ cells were counted in 3 consecutive sections from bregma −1.70 mm for each animal (18). To perform these analyses, 5 animals were used per group (SC, *n* = 5 and HFD, *n* = 5). The Image J Software (http://rsbweb.nih.gov/ij/) was used to automatically count the cells.

### ELISA to Determinate Cytokine Concentration in the Serum and Hypothalamus

Isolated hypothalamus and serum were immediately kept frozen (−80). Hypothalamus were homogenized in saline phosphate-buffered supplemented with protease inhibitors in a beadruptor^®^ at medium speed for 15 s. Then the samples were centrifuged at 11,000 rpm for 20 min (4°C) to remove the insoluble material, and only the supernatant was used for the assay. The concentrations of cytokines in hypothalamus and serum (TNF-α and IL-1β) in the supernatants were assessed by ELISA using the Duo Set kit (R&D System) and IL6 using IL-6 mouse ELISA kit (Thermofischer Scientific). The concentrations were normalized by the amount of protein in the samples, determined by the method described by Bradford.

### Statistical Analysis

Results are presented as the mean ± SEM. Data were analyzed using a Student's *t-*test following confirmation of normal distribution by the Kolmogorov–Smirnov test. One-way analysis of variance (ANOVA) followed by multiple comparisons between groups using Tukey's *post-hoc* test were used when differences among more than two groups were analyzed. The log-rank test was used for survival rate analysis. Statistical significance for all analyses was set at *p* < 0.05. All statistical comparisons were performed using STATISTICA (StatSoft Inc., Tulsa, OK, USA) and GraphPad Prism 6.01 (http://www.graphpad.com/scientific-software/prism/).

## Results

### Hypothalamic and Peripheral Inflammatory and Anti-inflammatory Profiles Following Short-Term HFD Consumption

**Figure 1:**
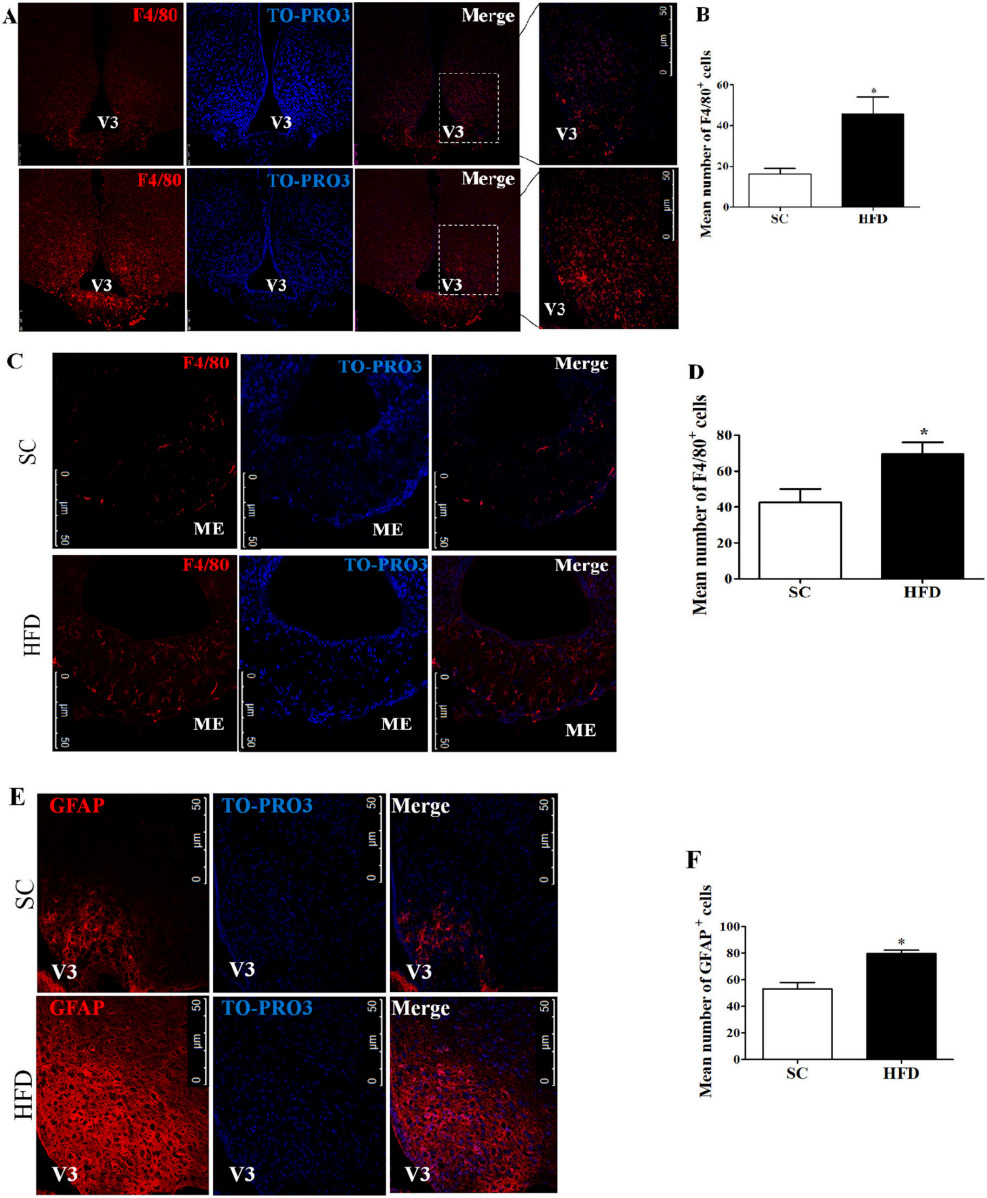
Hypothalamic inflammation following short-term HFD consumption. **(A)** Confocal images illustrating F4/80+ cells (red) and nuclear labeling with TO-PRO-3® (blue) in the ARC, in coronal brain sections (15-μm) from mice fed with standard chow (SC) or a high-fat diet (HFD) for 3 days; **(B)** Quantitation of the number of F4/80+ cells in the ARC of mice fed with SC or a HFD (SC, *n* = 5 and HFD, *n* = 5); **(C)** Confocal images illustrating F4/80+ cells (red) and nuclear labeling with TO-PRO-3® (blue) in the ME, in coronal brain sections (15-μm) from mice fed with SC or a HFD for 3 days; **(D)** Quantitation of the number of F4/80^+^ cells in the ME of mice fed with SC or a HFD (SC, *n* = 5 and HFD, *n* = 5); **(E)** Confocal images illustrating GFAP+ cells (red) and nuclear labeling with TO-PRO-3® (blue) in the ARC, in coronal brain sections (15-μm) from mice fed with SC or a HFD (3 days); **(F)** Quantitation of the number of GFAP+ cells in the ARC of mice fed with SC or a HFD (SC, *n* = 5 and HFD, *n* = 5). V3, third ventricle; ME, median eminence. The bars represent the mean ± SEM. ^*^Means significantly different as shown by unpaired *t-*tests (^*^p < 0.05). Scale bars: 50 μm.

Firstly, the hypothalamic inflammation in our experimental model was investigated. Immunofluorescence staining of F4/80 and GFAP was used to identify microglia and astrocytes, respectively. The number of F4/80+ cells in the arcuate nucleus (ARC) and median eminence (ME) (Figures 1A–D) was higher following short-term HFD consumption as compared with mice fed SC. Additionally, the number of GFAP+ cells in the arcuate nucleus also increased following short-term HFD consumption as compared with mice fed SC (Figures 1E,F).

**Figure 2:**
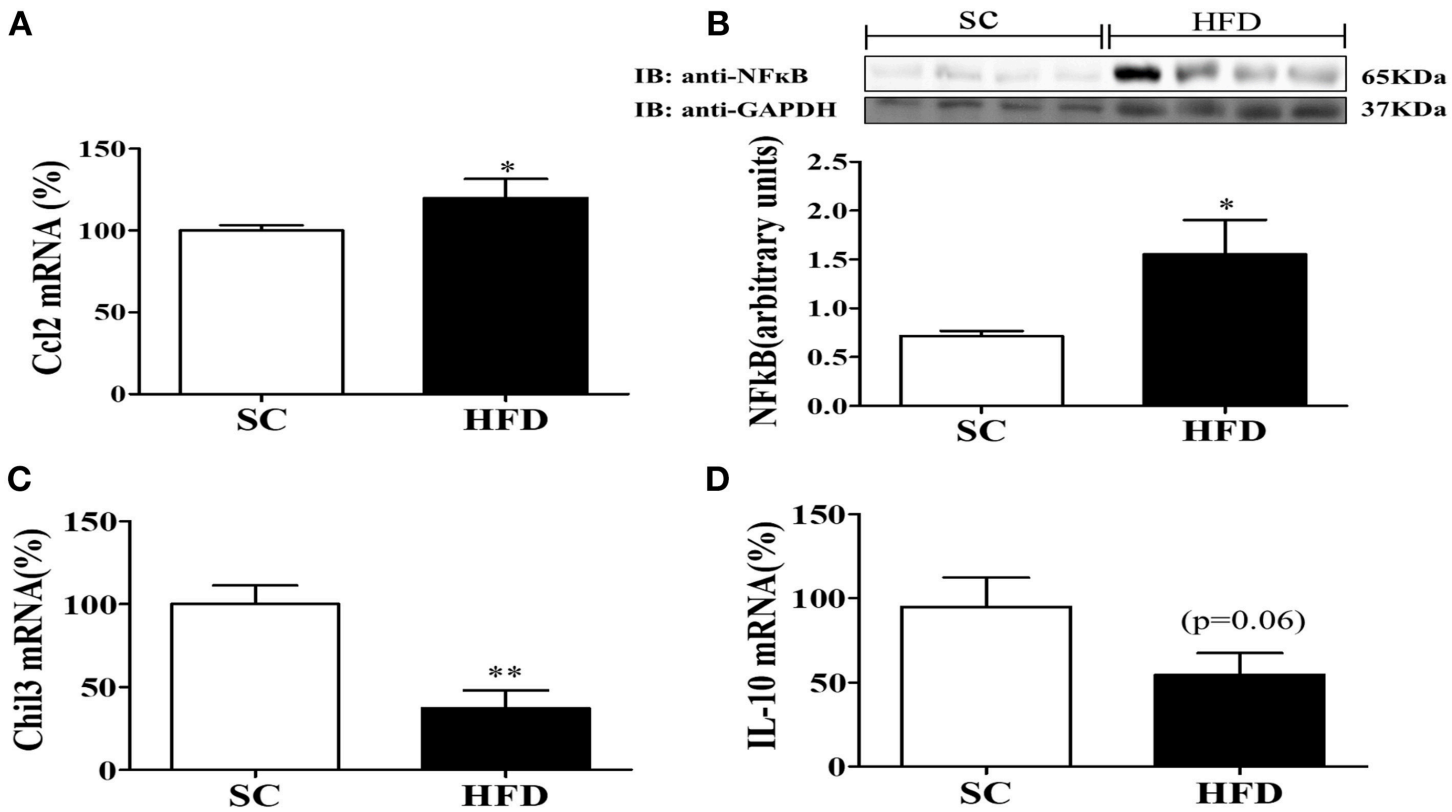
Anti-inflammatory and inflammatory markers in the hypothalamus following short-term HFD consumption. **(A)** mRNA expression levels of CCL2 (SC, *n* = 5 and HFD, *n* = 5); **(B)** Protein expression levels of NFκB (SC, *n* = 4 and HFD, *n* = 4); **(C)** mRNA expression levels of Chil3 (SC, *n* = 5 and HFD, *n* = 5), and; **(D)** mRNA expression levels of IL-10 (SC, *n* = 5 and HFD, *n* = 5); in the hypothalamus of mice fed SC or a HFD for 3 days. The bars represent the mean ± S.E.M. ^*^Means significantly different as shown by unpaired *t-*tests (^*^p < 0.05 and ^**^p < 0.01).

The hypothalamic mRNA levels of C-C chemokine ligand 2 (CCL2) were also assessed along with the protein levels of NFκB. The basal levels of hypothalamic CCL2 mRNA and NFκB protein were increased in mice fed a HFD as compared with mice fed SC (Figures 2A,B). Conversely, the mRNA levels of the hypothalamic anti-inflammatory markers, chitinase-like 3 (Chil3) and IL10, were reduced in HFD-fed mice as compared with SC-fed mice (Figures 2C,D) (*p* < 0.01 and *p* < 0.06, respectively).

**Figure 3:**
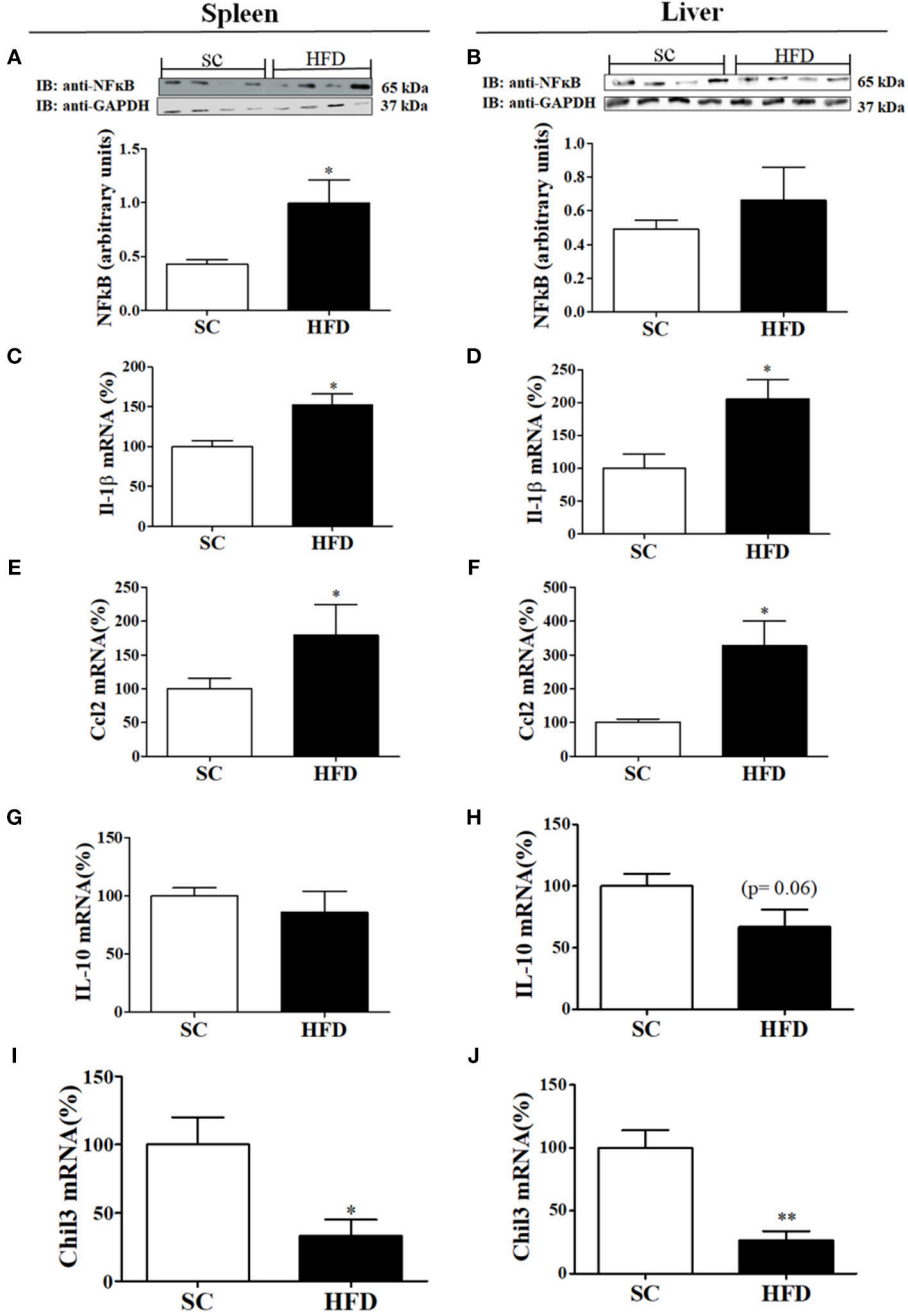
Anti-inflammatory and inflammatory markers in the spleen and liver following short-term HFD consumption. **(A,B)** Protein expression levels of NFκB (SC, *n* = 4 and HFD, *n* = 4) in the spleen **(A)** and liver **(B)**; **(C,D)** IL-1β mRNA expression levels in the spleen **(C)** and liver **(D)** (SC, *n* = 5 and HFD, *n* = 5); **(E,F)** CCL2 mRNA expression levels in the spleen **(E)** and liver **(F)** (SC, *n* = 5 and HFD, *n* = 5); **(G,H)** IL-10 mRNA expression levels in the spleen **(G)** and liver **(H)** (*n* = 5 and HFD, *n* = 5); **(I,J)** Chil3 mRNA expression levels in the spleen **(I)** and liver **(J)** (SC, *n* = 5 and HFD, *n* = 5) of SC-fed and HFD-fed mice. The bars represent the mean ± S.E.M. ^*^Means significantly different as shown by unpaired *t-*tests (^*^p < 0.05, ^**^p < 0.01).

Inflammatory and anti-inflammatory molecules in the spleen and liver of HFD-fed and SC-fed mice were also evaluated (Figure 3). In the liver, NFκB protein levels did not differ between HFD-fed and SC-fed mice; however, the NFκB protein levels in the spleen were significantly higher in mice fed a HFD than in mice fed SC (Figures 3A,B). IL1β and CCL2 mRNA levels were increased in HFD-fed mice as compared with SC-fed mice (Figures 3C–F). Liver mRNA levels of IL10 showed a tendency to be reduced in mice fed a HFD as compared with mice fed SC (*p* = 0.06); however, the levels in the spleen did not differ (Figures 3G,H). Moreover, in both the spleen and liver, Chil3 mRNA levels, a marker of M2 macrophages, were also reduced in HFD-fed mice as compared with SC-fed mice (Figures 3I,J).

### Short-Term HFD Consumption Exacerbates the Inflammatory Response to LPS

**Figure 4:**
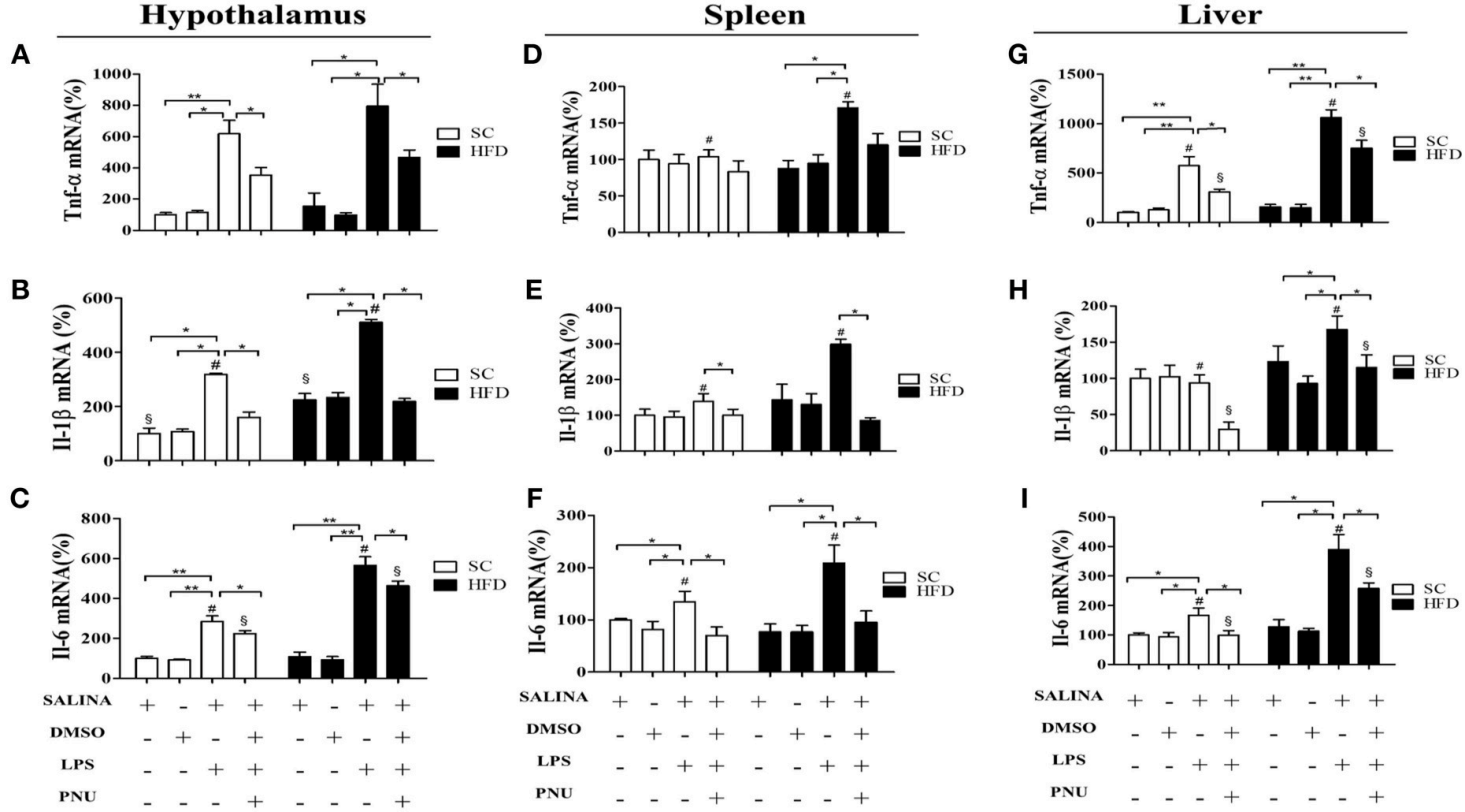
Cytokine levels in the hypothalamus and peripheral tissues following i.p. LPS challenge and i.p. PNU-282987 stimulation. **(A)** mRNA expression levels of TNFα (SC, *n* = 5 and HFD, *n* = 5); **(B)** mRNA expression levels of IL-1β (SC, *n* = 5 and HFD, *n* = 5); **(C)** mRNA expression levels of IL-6 (SC, *n* = 5 and HFD, *n* = 5); in the hypothalamus; **(D)** mRNA levels of TNFα (SC, *n* = 5 and HFD, *n* = 5); **(E)** mRNA expression levels of IL-1β (SC, *n* = 5 and HFD, *n* = 5); **(F)** mRNA expression levels of IL-6 (SC, *n* = 5 and HFD, *n* = 5) in the spleen; **(G)** mRNA expression levels of TNFα (SC, *n* = 5 and HFD, *n* = 5); **(H)** mRNA expression levels of IL-1β (SC, *n* = 5 and HFD, *n* = 5); **(I)** mRNA expression levels of IL-6 (SC, *n* = 5 and HFD, *n* = 5) in the liver; of mice fed SC or a HFD for 3 days and injected intraperitoneally with a high dose of LPS (12 mg/kg) followed by stimulation with PNU-282987 (3 mg/kg). The bars represent the mean ± S.E.M. ^*^Means significantly different as shown by *one-way ANOVA* (^*^p < 0.05; ^**^p < 0.01; #*p* < 0.05; §*p* < 0.05). # and § to compare SC and HFD.

To evaluate the susceptibility of the mice to inflammatory agents, we used intraperitoneally administered LPS to stimulate a hypothalamic inflammatory response. As expected, LPS increased the mRNA levels of TNFα, IL-1β, and IL-6 in the hypothalamus (Figures 4A–C), spleen (Figures 4D–F), and liver (Figures 4G–I) of both SC-fed and HFD-fed mice. However, the inflammatory effects of intraperitoneally administered LPS were more pronounced in HFD-fed mice as compared with SC-fed mice (Figure 4) for all cytokines evaluated, except to hypothalamic TNFα (Figure 4A). The capacity of α7nAChR to reduce inflammatory response was evaluated in both SC-fed and HFD-fed mice following i.p. injection of PNU-282987, a selective α7nAChR agonist. In both groups (SC-fed and HFD-fed), PNU-282987 reduced the mRNA levels of TNFα, IL-1β, and IL-6 in all tissues evaluated. However, the effect of PNU-282987 on hypothalamic IL-6 mRNA (Figure 4C) and hepatic TNFα, IL-1β, and IL-6 mRNA levels (Figures 4G–I) in HFD-fed mice was significantly lower.

**Figure 5:**
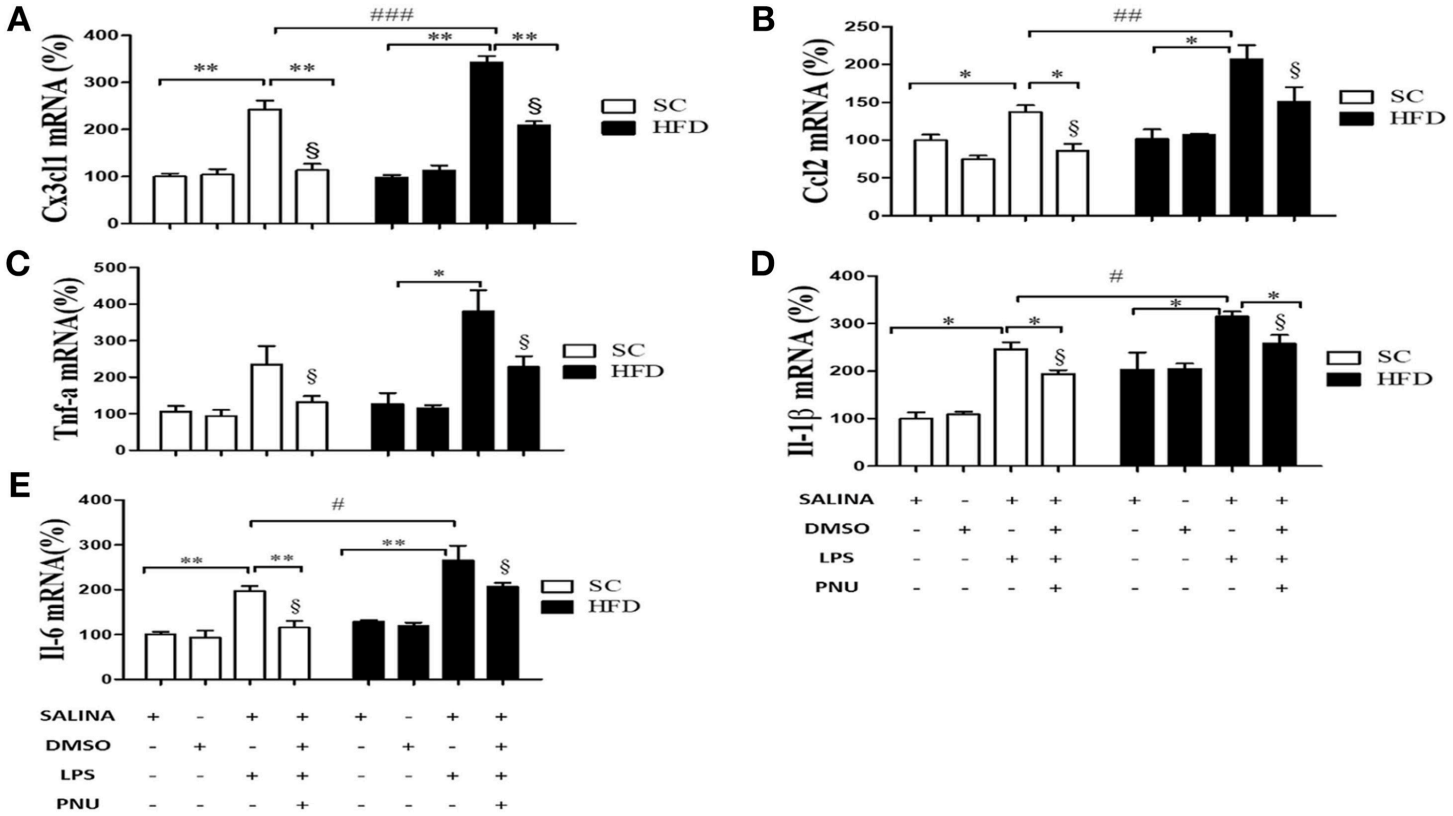
Chemokine and cytokine levels in the hypothalamus following i.p. LPS challenge and i.c.v. PNU-282987 stimulation. **(A)** mRNA expression levels of CX3CL1 (SC, *n* = 6 and HFD, *n* = 6); **(B)** mRNA expression levels of CCL2 (SC, *n* = 6 and HFD, *n* = 6); **(C)** mRNA expression levels of TNFα (SC, *n* = 5 and HFD, *n* = 5); **(D)** mRNA expression levels of IL-1β (SC, *n* = 6 and HFD, *n* = 6); and **(E)** mRNA expression levels of IL-6 (SC, *n* = 6 and HFD, *n* = 6) in the hypothalamus of mice fed SC or a HFD for 3 days and injected intraperitoneally with a high dose of LPS (12 mg/kg) followed by stimulation intracerebroventricularly with PNU-282987 (10 pmoles/mouse). The bars represent the mean ± S.E.M. *Means significantly different as shown by *one-way ANOVA* (*p < 0.05; **p < 0.01; ^#^*p* < 0.05; ^*##*^*p* < 0.001; ^*###*^*p* < 0,01; ^§^*p* < 0.05). #, ##, ### and §to compare SC and HFD.

Considering the role of the hypothalamus in homeostasis and the reduced hypothalamic expression of α7nAChR, additional analyses were performed following i.c.v injection of PNU-282987 (Figure 5). Initially, as expected, LPS injection increased hypothalamic mRNA levels of the chemokines CX3CL1 and CCL2 and cytokines IL-1β and IL-6 in both groups (SC-fed and HFD-fed). Besides, the effect of LPS was more pronounced in HFD-fed compared to SC-fed mice for most of the inflammatory markers evaluated. When the analyses were performed following i.c.v injection of PNU-282987, hypothalamic mRNA levels of the chemokines CX3CL1 and CCL2 and cytokines IL-1β, TNFα, and IL-6 were significantly reduced in SC-fed and HFD-fed mice. However, for all inflammatory markers evaluated (CX3CL1, CCL2, IL-1β, IL-6, and TNFα) the mRNA levels were significantly higher in HFD-fed than SC-fed mice (Figure 5).

**Figure 6:**
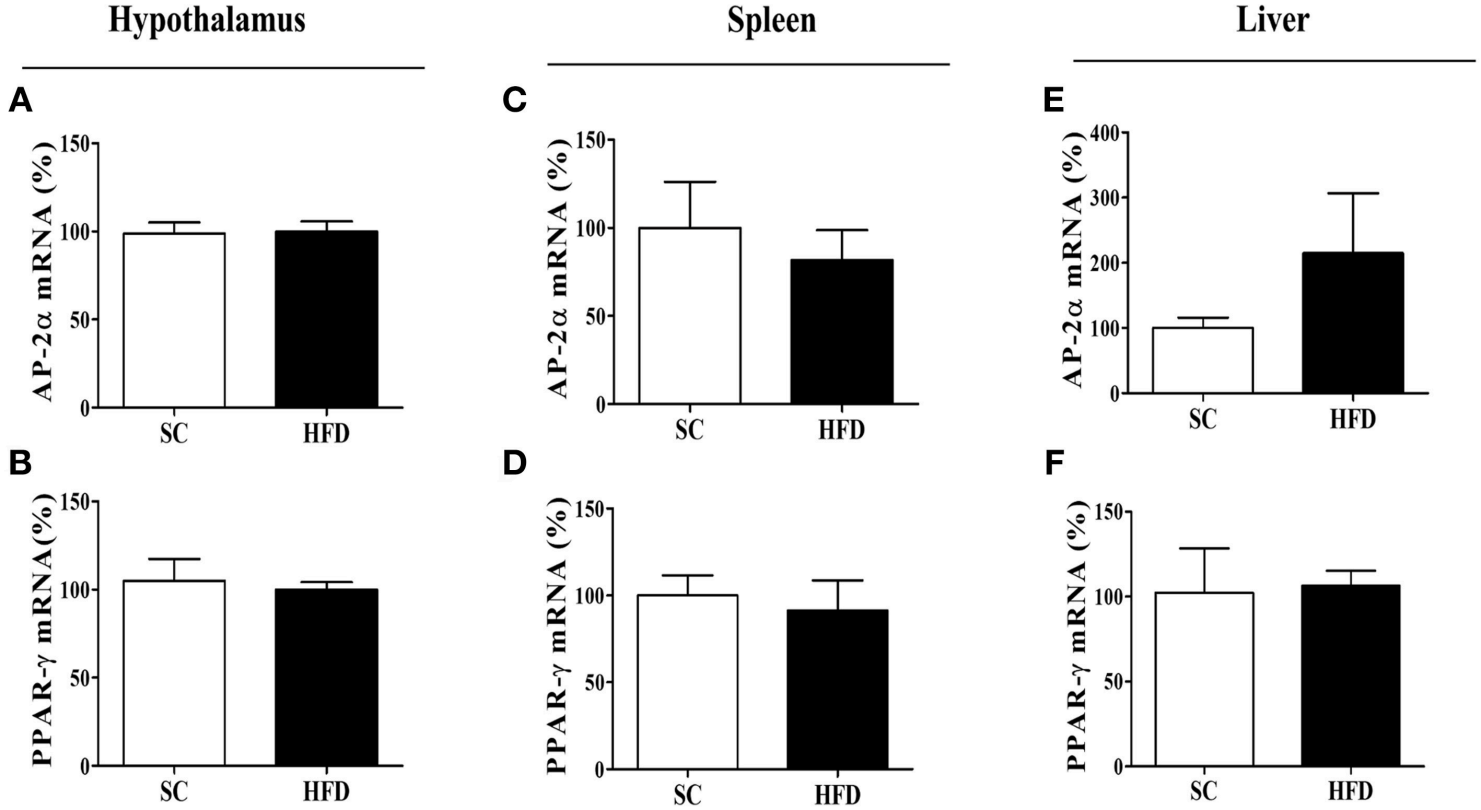
mRNA expression levels of CHRNA7 transcription factors following HFD consumption. The mRNA expression levels of AP-2α **(A)** in the hypothalamus (SC, *n* = 5 and HFD, *n* = 5), **(B)** spleen (SC, *n* = 5 and HFD, *n* = 5), and **(C)** liver (SC, *n* = 5 and HFD, *n* = 5); the mRNA levels of PPAR-γ **(D)** in the hypothalamus (SC, *n* = 5 and HFD, *n* = 5), **(E)** spleen (SC, *n* = 5 and HFD, *n* = 5), and **(F)** liver (SC, *n* = 5 and HFD, *n* = 5) of mice fed SC or a HFD for 3 days. The bars represent the mean ± SEM.

Additionally, we evaluated the level of cytokines in the hypothalamus (TNFα, IL-1β, and IL-6) and serum (TNFα and IL-1β) (Figure S1) using ELISA method. As can be observed in the supplementary figures the cytokine levels in the hypothalamus and serum were not significantly modified by LPS treatment. Serum IL-1β was an exception considering that in SC mice IL-1β the level increased significantly after LPS treatment and PNU reduced the level of this cytokine. Unexpectedly in HFD mice the effect of LPS treatment was significantly lower than SC mice. To perform all ELISA analyses, we used the same protocol previously used to evaluate mRNA levels in hypothalamus and we believe that the exposure time to LPS and PNU was not enough to detect differences in protein level of these cytokines.

In addition, another sepsis model was used to show higher susceptibility to hypothalamic inflammation in mice fed HFD as compared with mice fed SC. Following CLP surgery, the hypothalamic mRNA levels of CCL2, CX3CL1, IL-1β, TNFα, and IL-6 were significantly increased in HFD-fed mice as compared with SC-fed mice. Conversely, IL-10 and Chil3 mRNA levels were reduced in mice fed a HFD as compared with mice fed SC, suggesting damage to the inflammatory response control mechanism (Figures 9A–G).

### Expression of the Nicotinic Acetylcholine Receptor α7 Subunit (α7nAChR)

**Figure 7:**
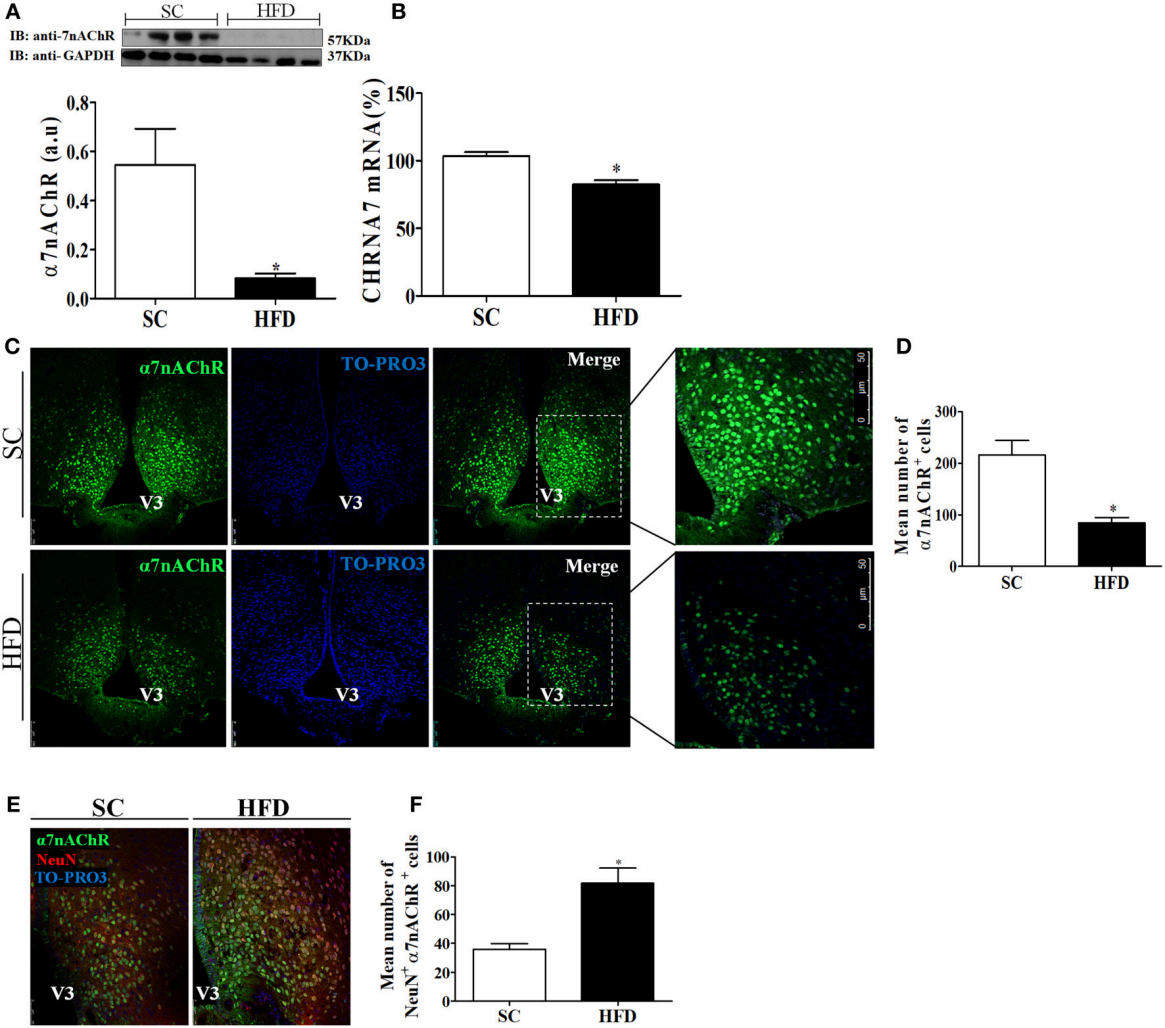
CHRNA7 expression levels in the hypothalamus following HFD consumption. **(A)** The protein expression levels of α7nAChR (SC, *n* = 4 and HFD, *n* = 4); and **(B)** The mRNA expression levels of α7nAChR in the hypothalamus of mice fed SC or a HFD for 3 days (SC, *n* = 5 and HFD, *n* = 5); **(C)** Confocal images illustrating α7nAChR+ cells (green) and nuclear labeling with TO-PRO-3® (blue) in coronal brain sections (15-μm) from mice fed SC or a HFD for 3 days. **(D)** Quantitation of the number of α7nAChR+ cells in the ARC of mice fed SC or a HFD (SC, *n* = 5 and HFD, *n* = 5). **(E)** Confocal images illustrating α7nAChR+ cells (green), NeuN+ cells (red), and nuclear labeling with TO-PRO-3® (blue) in coronal brain sections (15-μm) from mice fed SC or a HFD for 3 days. **(F)** Quantitation of the number of α7nAChR+/NeuN+ cells in the ARC of mice fed SC or HFD. V3, third ventricle. The bars represent the mean ± SEM. ^*^Means significantly different as shown by unpaired *t-*tests (^*^p < 0.05). Scale bars: 50 μm.

Considering the role of α7nAChR in the anti-inflammatory response controlling inflammatory cytokine production, we evaluated the expression of this receptor subunit in the hypothalamus, liver, and spleen of mice fed SC or HFD. Short-term HFD consumption markedly reduced both the protein and mRNA levels of α7nAChR in the hypothalamus (Figures 7A,B). To assess whether hypothalamic arcuate nucleus expression of α7nAChR was affected by short-term HFD consumption, we performed an immunofluorescence analysis in coronal sections. The number of α7nAChR+ cells in the arcuate nucleus was significantly diminished in HFD-fed mice as compared with SC-fed mice. While in SC m mice the α7nAChR+ cells represent 15% of total of cells evaluated in HFD mice only 8.2% were α7nAChR+ cells (Figures 7C,D). Additionally, the immunofluorescence analysis was performed to identify NeuN+ cells that also express α7nAChR protein. As can be observed in the Figures 7E,F the number of mature neurons that express α7nAChR protein was significantly higher in HFD than SC mice. In SC mice 3.7% of cells evaluated were NeuN+/α7nAChR+ cells while in HFD mice the percentage was 8.7%. Besides, we performed the western blot analysis of pSTAT3 and STAT3 protein in the hypothalamus of mice fed SC or HFD. Basal phosphorylation of STAT3 was reduced in HFD-fed mice as compared to the SC-fed mice, but total STAT3 in the hypothalamus was increased in HFD-mice (Figure S2).

**Figure 8:**
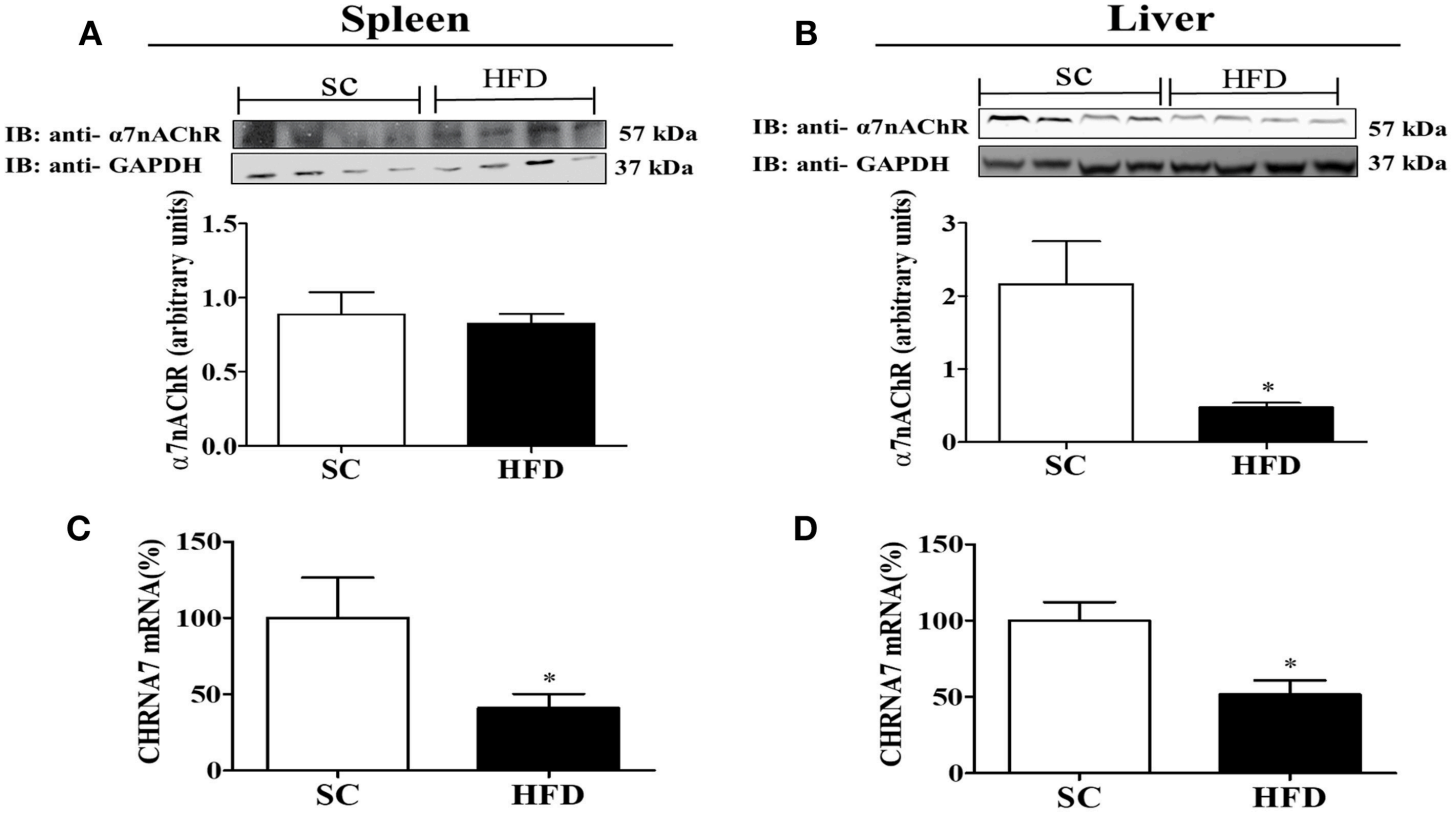
α7nAChR and CHRNA7 expression levels in the spleen and liver following HFD consumption. **(A,B)** Protein expression levels of α7nAChR in the **(A)** spleen and **(B)** liver of mice fed SC or a HFD for 3 days (SC, *n* = 4 and HFD, *n* = 4); **(C,D)** α7nAChR mRNA expression levels in the spleen **(C)** and liver **(D)** of SC-fed and HFD-fed mice (SC, *n* = 4 and HFD, *n* = 4). The bars represent the mean ± SEM. ^*^Means significantly different as shown by unpaired *t*-tests (^*^p < 0.05).

Furthermore, the expression levels of α7nAChR in the spleen and liver were evaluated following short-term HFD consumption (Figure 8). Although α7nAChR mRNA levels were significantly reduced (*p* < 0.05) in the spleen and liver after HFD consumption, the protein levels of α7nAChR were only reduced in the liver (*p* < 0.05). Interestingly, differently from that observed in the hypothalamus, splenic levels of stat3 and pSTAT3 seems to increase in mice fed HFD (Figure S2), suggesting that this pathway was not affected by HFD consumption.

**Figure 9:**
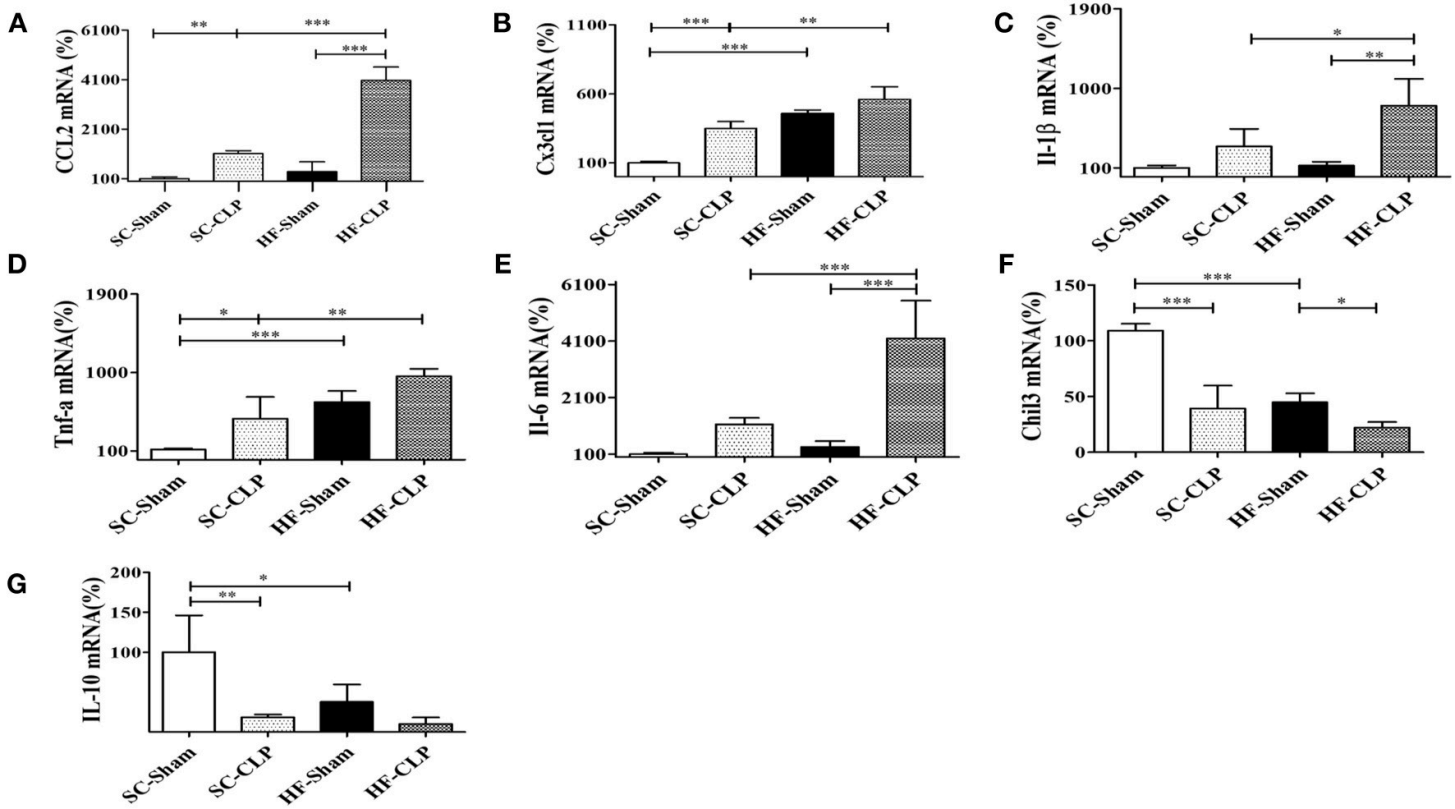
Chemokine and cytokine levels in the hypothalamus following sepsis induction by CLP. **(A)** mRNA expression levels of CCL2 (SC, *n* = 5 and HFD, *n* = 5); **(B)** mRNA expression levels of CX3CL1 (SC, *n* = 6 and HFD, *n* = 6); **(C)** mRNA expression levels of IL-1β (SC, *n* = 6 and HFD, *n* = 6); **(D)** mRNA expression levels of TNFα (SC, *n* = 6 and HFD, *n* = 6); **(E)** mRNA expression levels of IL-6 (SC, *n* = 6 and HFD, *n* = 6); **(F)** mRNA expression levels of Chil3 (SC, *n* = 6 and HFD, *n* = 6); and **(G)** mRNA expression levels of IL-10 (SC, *n* = 6 and HFD, *n* = 6) in the hypothalamus of mice fed SC or a HFD for 3 days followed by induction of sepsis by cecal ligation and puncture surgery. The bars represent the mean ± SEM. ^*^Means significantly different as shown by *one-way ANOVA* (^*^p < 0.05; ^**^p < 0.01; ^***^p < 0.0001).

The mRNA levels of AP-2α and PPARγ, important transcription factors that regulate the α7nAChR gene in the hypothalamus, were evaluated in the spleen and liver of SC-fed and HFD-fed mice. For both transcripts (AP-2α and PPARγ), we found no difference in levels between groups (Figures 6A–F).

### Short-Term HFD-Fed Mice Are More Susceptible to the Development of Sepsis and Subsequent Death Following LPS Challenge or CLP Surgery

**Figure 10:**
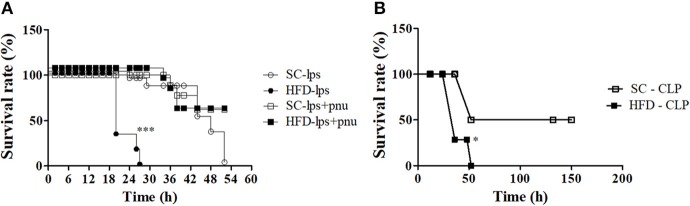
Survival rate following LPS challenge and CLP surgery of SC-fed and HFD-fed mice. Mice were fed SC or a HFD for 3 days. Subsequently, sepsis was induced by two different methods. **(A)** A lethal dose of LPS (30 mg/kg) was administered via intraperitoneal injection in mice the received icv injection of PNU (10 pmoles/mouse) (SC-LPS, *n* = 7 opened circles, HFD-LPS, *n* = 5 closed circles, SC-lps+pnu, *n* = 5 opened squares, and HFD-lps+pnu, *n* = 5 closed squares); or **(B)** Cecal ligation and puncture surgery was performed (SC-CLP, *n* = 5 opened squares and HFD-CLP, *n* = 5 closed squares). The survival rate was recorded every 2 h. The results represent the mean ± S.E.M. **(A)** ^***^p < 0.0001 HFD-lps vs SC-lps, **(B)** ^*^p < 0.05 HFD-CLP vs SC-CLP (log-rank test).

α7nAChR plays an important role in the reduction of sepsis severity. To identify whether mice fed a HFD are more prone to developing sepsis, the survival curves of SC-fed and short-term HFD-fed mice were compared following either a lethal dose of LPS (30 mg/kg; i.p.) or CLP surgery. Short-term HFD consumption significantly reduced the survival after LPS challenge or CLP surgery, and HFD-fed mice died significantly quicker (*p* < 0.001) as compared with SC-fed mice (Figures 10A,B). Interestingly, PNU icv injection recovered the ability to respond to challenges with LPS (Figure 10A).

## Discussion

In the current study, we provide comprehensive results regarding the harmful effects of short-term HFD consumption on the inflammatory response in the hypothalamus and peripheral tissues.

Initially, we showed increased immunostaining of F4/80+ cells (microglial/macrophage marker) in the arcuate nucleus (ARC) and median eminence (ME) of HFD-fed mice. Moreover, HFD-fed mice showed a higher number of GFAP+ cells (astrocyte activation marker) in the ARC than did SC-fed mice. Furthermore, the expression levels of pro-inflammatory markers in the hypothalamus, liver, and spleen of HFD-fed mice were also increased, while the expression levels of anti-inflammatory markers were diminished, as compared with SC-fed mice. These results suggest that rapid onset of hypothalamic inflammation may be associated with glial responses. Similar results have been described by Thaler and collaborators in rats (4). It is known that the medial basal hypothalamus (MBH), including the ARC and ME, is sensitive to saturated fatty acids (SFA) and is responsible for controlling food intake and intermediate metabolism (4, 19). In addition, the blood-brain barrier surrounding the MBH region, which is composed of fenestrated vessels that facilitate the infiltration of macrophages and the activation of microglia by SFA, has also been shown to be associated with neuronal stress in the MBH region (20). Recently, a study performed by the same research group showed that microglial inflammatory activation orchestrates a multicellular hypothalamic response that mediates susceptibility to obesity (21).

Despite breakthroughs in medical science, sepsis remains a leading cause of death in hospitals. Studies have shown that the uncontrolled production of pro-inflammatory cytokines, such as TNF-α and IL-1β, plays a key role in the development of sepsis (22, 23). Increased sensitivity of HFD-fed mice to LPS (24, 25), as well as increased propensity to develop sepsis, has also been reported (24, 26). Recently, De Martini et al. (27) demonstrated that obese mice have a greater susceptibility to sepsis and a higher mortality rate; however, the mechanism of sepsis induction following short-term HFD consumption and challenge with LPS has not yet been established. Here, we show that short-term HFD consumption had an important impact on the susceptibility to sepsis induced by LPS challenge or CLP surgery.

The present results show that HFD-fed HFD mice were more likely to develop central and systemic inflammation following LPS challenge or CLP surgery, as demonstrated by increased cytokine and chemokine levels and a low survival rate. These effects are likely associated with diminished α7nAChR expression and STAT3 activation. α7nAChR is known to inhibit cytokine production by tyrosine kinase Jak2 recruitment and STAT3 phosphorylation, which binds to NFκB and inhibits nuclear translocation, subsequently inhibiting TNF-α production (8, 28). Additionally, Vachharajani and others have shown that sirtuins (Sirt) have an important role in the control of inflammation. SIRT1 and SIRT6 are nuclear sirtuins that can deacetylate RelA/p65 promoting its proteasome degradation and consequently the reduction of cytokines levels (29, 30). Besides, SIRT1 and SIRT6 regulate the metabolic switch from glycolysis to fatty acid oxidation enabling the adaptation to acute inflammation and M2 macrophage response (31). In this context, Vachharajani et al. show that the SIRT1 deficiency observed in ob/ob mice exacerbates the response to sepsis (32). We evaluated SIRT1 expression in SC and short term HFD mice (Figure S3) and, interestingly, HFD consumption seems to reduce hypothalamic SIRT1 levels compared to SC mice. On the other hand, the icv injection of PNU was able to increase SIRT1 expression in SC mice but not in HFD mice, suggesting that there is an interaction between α7nAChR receptor pathway and SIRT1 expression. As discussed before, SIRT1 inhibition can be an additional mechanism associated to exacerbated inflammatory response induced by HFD consumption. Damage to SIRT activity can impair metabolic programming and lead to both increased activity of hypothalamic NFKB pathway and susceptibility to sepsis (29, 30).

To characterize the nicotinic cholinergic anti-inflammatory response, the α7nAChR expression levels in central and peripheral tissues were evaluated. α7nAChR protein and mRNA levels in the hypothalamus of HFD-fed mice were reduced as compared with SC-fed mice. Furthermore, immunofluorescence analysis indicates a marked reduction in α7nAChR+ cells in the hypothalamus of mice fed a HFD. Interestingly, the number of neuronal cells (NeuN+) expressing α7nAChR increased in HFD-mice compared to SC-mice, suggesting that α7nAChR expression in neurons was stimulated by HFD consumption. Additional experiments are necessary to understand if the microglia and astrocytes can be differently affected by HFD consumption compared to neurons. Similarly, lower levels of α7nAChR protein and mRNA were also observed in the liver of HFD-fed mice as compared with SC-fed mice. Conversely, α7nAChR protein levels in the spleen were not different; however, α7nAChR mRNA levels were significantly lower in HFD-fed mice than in SC-fed mice. Considering the fundamental function of the spleen in the inflammatory response it is possible that the cholinergic receptors in this organ are protected from posttranslational mechanisms that signal for protein degradation. In addition, the hypothalamus shows faster activation of inflammatory pathways than peripheral tissues after consumption of HFD for a short period of time, as demonstrated by other studies (4, 5). We do not rule out that the activation of inflammatory pathways is directly related to the increase in receptor degradation. Although it has not been evaluated in this study, an interesting area to be investigated is the relationship between the inflammatory pathways and the activation of posttranscriptional and translational mechanisms that affect receptor expression. Thus, here we demonstrate that short-term HFD consumption adversely affected α7nAChR expression in tissues important in homeostasis control.

α7nAChR plays a key role in both central and peripheral mechanisms involved in energy homeostasis and control of the inflammatory response, and is considered a critical component of the innate immune system (33, 34). Wang et al. (7) showed a role of the α7 subunit in the cholinergic anti-inflammatory pathway during obesity, inflammation, and insulin resistance. The researchers demonstrated that the activation of the cholinergic anti-inflammatory pathway suppresses inflammation in adipose tissue and improves insulin sensitivity in animal models with diet-induced obesity (DIO) and genetic obesity (db/db); however, animals that do not express α7nAChR (α7KO) present abnormal inflammation and decreased insulin sensitivity. Thus, the activation of the cholinergic anti-inflammatory pathway may play a protective role against obesity-induced inflammation and insulin resistance.

The main physiological mechanism associated with α7nAChR activation has been shown to be promotion of anti-inflammatory effects (35). This effect is mediated by the cholinergic anti-inflammatory pathway through the vagus nerve and ACh release that acts on cholinergic receptors, such as α7nAChR in macrophages, suppressing the release of TNF-α and other pro-inflammatory cytokines (36). Thus, the diminished expression of α7nAChR likely increases the activation of NFκB and leads to the release of inflammatory cytokines and recruitment of inflammatory cells (37). Therefore, the reduction in α7nAChR expression could render the tissue more susceptible to inflammatory processes and may explain the increased mortality rate observed in mice following short-term HFD consumption, as demonstrated here. Besides, the mortality was reduced by the i.c.v. injection of PNU, reinforcing the importance of hypothalamic α7nAChR to the systemic inflammatory response.

In this context, the polarization of M1 and M2 macrophages can be altered and imbalanced, consistent with a previous study by St-Pierre and collaborators (38), in which the involvement of nicotinic acetylcholine receptors, particularly the α7 subunit, in the control of proliferation and polarization of M1-type immune cells was demonstrated. In an interesting study by Wang et al. (39), mice that did not express PTEN phosphatase in RIP neurons in the hypothalamus had greater anti-inflammatory reflex activation, which is associated with phenotypic stimulation of macrophages polarizing to M2 type.

To explore this possibility and the impairment of α7nAChR signaling, PNU-282987 (a selective α7nAChR agonist) was administered peripherally or centrally after LPS challenge, and STAT3 activation and cytokine production were analyzed. Firstly, the analyses performed after intraperitoneal injection of PNU-282987 showed that α7nAChR activation was sufficient to abrogate the effects of LPS in SC-fed and HFD-fed mice. However, the PNU-282987 was more efficient to inhibit hepatic TNF-α, IL-1β, and IL-6 expression in SC-fed than HFD-fed mice. On the other hand, in the hypothalamus only IL-6 mRNA level presented similar effect, but in the spleen the PNU administration resulted in a similar effect in SC-fed and HFD-mice. As demonstrated here α7nAChR protein level in the spleen was similar between SC-fed and HFD-fed mice that could explain the similar effect of PNU-282987 in both groups. Besides, the α7nAChR receptor expressed in peripheral tissue (7) can be activated by PNU-282987 administered peritoneally. This effect can modulate systemic level of inflammatory cytokines and its effects on hypothalamus.

To isolate the participation of hypothalamic α7nAChR, the i.c.v. injection of PNU-282987 was performed. The effect of PNU-282987 is significantly lower in mice fed HFD than in mice fed SC, suggesting an impairment in cholinergic signaling by short-term HFD consumption. This is not surprising since hypothalamic α7nAChR expression was diminished in HFD-fed compared to SC-fed mice. Interestingly, although hypothalamic α7nAChR activation has not been associated with the cholinergic anti-inflammatory response, its expression appears to be important in the control of the intensity of local inflammation and consequently prevent metabolic disturbs associated to activation of inflammatory pathways in the hypothalamus (2, 4). To our knowledge there are no studies showing hypothalamic α7nAChR controlling central inflammatory response.

We suggest that the effect of HFD consumption on α7nAChR expression is an early effect of saturated fatty acids on the cell, i.e., prior to pro-inflammatory pathway activation. This first insult of fatty acids damages the cholinergic anti-inflammatory pathway, enabling the rise of cytokine expression and action on cellular components.

Although the research has reached its aim, there were some unavoidable limitations. First, we were not able to differentiate the importance of the α7nAChR expressed in neurons and microglia for the observed effects. In neurons α7nAChR activation could modulate both neuronal excitability and inhibit the expression of inflammatory cytokines. Second, we also believe that it would be important to identify the molecular mechanism related to the effect of short-term HFD with the reduction of α7nAChR expression. Finally, considering the difference in the immunological response between males and females it would also be interesting to evaluate the effect of HFD on α7nAChR expression and inflammatory response in females as well. Males and females (human and mice) present important differences in innate immunity. In human, TLR4 and TNF expression in neutrophils is higher in males than females (40). Similarly, in peritoneal macrophages from male mice the expression of TLR4 is higher than peritoneal macrophages from female (41). Besides the stimulation with LPS result in in greater pro-inflammatory cytokine production by immune cells from male than female mice (42). The differences were elegantly reviewed by Klein and Flanagan (43).

However, this study contributes to the better understanding of the effect of short-term HFD on the inflammatory response. This could be particularly important in pre-surgical recommendations. From this study important questions arise, such as whether the effect on the receptor is permanent, whether other tissues also exhibit similar behavior, and finally whether other molecular mechanisms may be associated with reduced tissue inflammation.

## Conclusion

The current experimental research demonstrates that short-term high-fat diet consumption increases mortality and organ injury following LPS-induced sepsis. These effects appear to occur through the impairment of the α7-mediated cholinergic anti-inflammatory response and result in an exacerbated inflammatory response.

## Ethics Statement

All applicable international, national, and/or institutional guidelines for the care and use of animals were followed.

## Author Contributions

AS, CS, CA, and SL performed most of the experiments and contributed to the data analysis. LS and AS performed CLP surgery and analysis. AS, CA, and MT wrote the original draft. AT and MM provided assistance to article supervision and discussion of results. MM, AT, and MT helped with funding acquisition. All the authors approved the final manuscript.

### Conflict of Interest Statement

The authors declare that the research was conducted in the absence of any commercial or financial relationships that could be construed as a potential conflict of interest.

## Supplementary Material

Figure S1 Hypothalamic **(A–C)** and serum cytokine levels **(D,E)**. Quantitative Elisa **(A)**, TNF-α, **(B)**, Il-1β and **(C)**. IL6 levels in hypothalamus and **(D)**, TNF-α and **(E)**. Il-1β levels in serum of mice injected intraperitoneally with a high dose of LPS (12 mg/kg) followed by stimulation intracerebroventricularly with PNU-282987 (10 pmoles/mouse). Significant differences between individual groups are shown (ANOVA with *post-hoc* Tukey HSD test: ^*^*p* < 0.05).

Figure S2 Protein content of pSTAT3 and STAT3 in the hypothalamus **(A,C)** and spleen **(B,D)** STAT3. Mice were fed standard chow diet (SC) or high fat diet (HFD) for 3 days. The bars represent the mean S.E.M. *n* = 3–4. ^*^Means significantly different by unpaired *t-*tests (^*^*p* < 0.05).

Figure S3 Hypothalamic SIRT1 level. Protein content of Sirt1 (SC, *n* = 3 and SC-PNU, *n* = 3; HFD, *n* = 3 and HFD-PNU, *n* = 3) in the hypothalamus of mice fed standard chow diet (SC) or high fat diet (HFD) for 3 days and/or received icv injection of the PNU (10 pmoles/mouse). The bars represent the mean S.E.M. ^*^Means significantly different by ANOVA with *post-hoc* Tukey HSD test: ^*^*p* < 0.05.

## Abbreviations

μm: Micrometer
Ach: Acetylcholine
AP2α: Transcription factor AP-2α
ARC: Arcuate nucleus
CCL2: C-C motif chemokine ligand 2
Chil3: Chitinase-like 3
CHRNA7: Neuronal acetylcholine receptor subunit α7
CLP: Cecal ligation and puncture
CNS: Central nervous system
CX3CL1: C-X3-C motif chemokine ligand 1
Cy3: Cyanine 3
DMSO: Dimethylsulfoxide
EM: Median eminence
F4/80: A glycoprotein expressed by murine macrophages
GAPDH: Glyceraldehyde-3-phosphate dehydrogenase
GFAP: Glial fibrillary acidic protein
HFD: High fat diet
HMB: Medial basal hypothalamus
I.c.v.: Intracerebroventricular
I.p.: Intraperitoneal
Il-1β: Interleukin 1β
Il-6: Interleukin 6
Il-10: Interleukin 10
LPS: Lipopolysaccharide
NeuN: Neuronal nuclei antigen
NF-B: Nuclear factor kappa B
PCR: Polymerase chain reaction
PFA: Paraformaldehyde
PNU: Positive allosteric modulator of the α7 subtype of neuronal nicotinic acetylcholine receptor
pSTAT3: Signal transducer and activator of transcription 3
SC: Standard chow
SEM: Standard error of the mean
TLR4: Toll-like receptor 4
TNF: Tumor necrosis factor
V3: Third ventricle.

## References

[1] Shi H Kokoeva MV Inouye K Tzameli I Yin H Flier JS. TLR4 links innate immunity and fatty acid – induced insulin resistance. J Clin Invest. (2006) 116:3015–25. 10.1172/JCI28898 17053832 PMC1616196

[2] Milanski M Degasperi G Coope A Morari J Denis R Cintra DE. Saturated fatty acids produce an inflammatory response predominantly through the activation of TLR4 signaling in hypothalamus: implications for the pathogenesis of obesity. J Neurosci. (2009) 29:359–70. 10.1523/JNEUROSCI.2760-08.2009 19144836 PMC6664935

[3] Cox AJ West NP Cripps AW. Obesity, inflammation, and the gut microbiota. Lancet Diabetes Endocrinol. (2015) 3:207–15. 10.1016/S2213-8587(14)70134-2 25066177

[4] Thaler JP Yi CX Schur EA Guyenet SJ Hwang BH Dietrich MO. Obesity is associated with hypothalamic injury in rodents and humans. J Clin Invest. (2012) 122:153–62. 10.1172/JCI59660 22201683 PMC3248304

[5] Waise TMZ Toshinai K Naznin F NamKoong C Md Moin AS Sakoda H. One-day high-fat diet induces inflammation in the nodose ganglion and hypothalamus of mice. Biochem Biophys Res Commun. (2015) 464:1157–62. 10.1016/j.bbrc.2015.07.097 26208455

[6] Luyer MD Greve JWM Hadfoune M Jacobs JA Dejong CH Buurman WA. Nutritional stimulation of cholecystokinin receptors inhibits inflammation via the vagus nerve. J Exp Med. (2005) 202:1023–9. 10.1084/jem.20042397 16216887 PMC2213207

[7] Wang H Yu M Ochani M Amella CA Tanovic M Susarla S. Nicotinic acetylcholine receptor α7 subunit is an essential regulator of inflammation. Nature. (2002) 421:384–8. 10.1038/nature01339 12508119

[8] de Jonge WJ van der Zanden EP The FO Bijlsma MF van Westerloo DJ Bennink RJ. Stimulation of the vagus nerve attenuates macrophage activation by activating the Jak2-STAT3 signaling pathway. Nat Immunol. (2005) 6:844–51. 10.1038/ni1229 16025117

[9] Kawashima K Yoshikawa K Fujii YX Moriwaki Y Misawa H. Expression and function of genes encoding cholinergic components in murine immune cells. Life Sci. (2007) 80:2314–9. 10.1016/j.lfs.2007.02.036 17383684

[10] The FO Cailotto C Van Der Vliet J De Jonge WJ Bennink RJ Buijs RM. Central activation of the cholinergic anti-inflammatory pathway reduces surgical inflammation in experimental post-operative ileus. Br J Pharmacol. (2011) 163:1007–16. 10.1111/j.1476-5381.2011.01296.x 21371006 PMC3130947

[11] The FO Boeckxstaens GE Snoek SA Cash JL Bennink R LaRosa GJ. Activation of the cholinergic anti-inflammatory pathway ameliorates postoperative ileus in mice. Gastroenterology. (2007) 133:1219–28. 10.1053/j.gastro.2007.07.022 17919496

[12] Wang X Yang Z Xue B Shi H. Activation of the cholinergic antiinflammatory pathway ameliorates obesity-induced inflammation and insulin resistance. Endocrinology. (2011) 152:836–46. 10.1210/en.2010-0855 21239433 PMC3040050

[13] Chunchai T Samniang B Sripetchwandee J Pintana H Pongkan W Kumfu S. Vagus nerve stimulation exerts the neuroprotective effects in obese-insulin resistant rats, leading to the improvement of cognitive function. Sci Rep. (2016) 6:26866. 10.1038/srep26866 27226157 PMC4880928

[14] Sun Y Li Q Gui H Xu D-P Yang Y-L Su D-F. MicroRNA-124 mediates the cholinergic anti-inflammatory action through inhibiting the production of pro-inflammatory cytokines. Cell Res. (2013) 23:1270–83. 10.1038/cr.2013.116 23979021 PMC3817544

[15] Payolla TB Lemes SF de Fante T Reginato A Mendes da Silva C de Oliveira Micheletti T. High-fat diet during pregnancy and lactation impairs the cholinergic anti-inflammatory pathway in the liver and white adipose tissue of mouse offspring. Mol Cell Endocrinol. (2016) 422:192–202. 10.1016/j.mce.2015.12.004 26687064

[16] Benatti RO Melo AM Borges FO Ignacio-Souza LM Simino LAP Milanski M. Maternal high-fat diet consumption modulates hepatic lipid metabolism and microRNA-122 (miR-122) and microRNA-370 (miR-370) expression in offspring. Br J Nutr. (2014) 111:2112–22. 10.1017/S0007114514000579 24666709

[17] Melo AM Benatti RO Ignacio-Souza LM Okino C Torsoni AS Milanski M. Hypothalamic endoplasmic reticulum stress and insulin resistance in offspring of mice dams fed high-fat diet during pregnancy and lactation. Metabolism. (2014) 63:682–92. 10.1016/j.metabol.2014.02.002 24636055

[18] George Paxinos KF Paxinos and Franklin's the Mouse Brain in Stereotaxic Coordinates. São Paulo: Academic Press (2012). p. 360 Available online at: https://www.elsevier.com/books/paxinos-and-franklins-the-mouse-brain-in-stereotaxic-coordinates/paxinos/978-0-12-391057-8

[19] Lam TKT Schwartz GJ Rossetti L. Hypothalamic sensing of fatty acids. Nat Neurosci. (2005) 8:579–584. 10.1038/nn1456 15856066

[20] Valdearcos M Robblee MM Benjamin DI Nomura DK Xu AW Koliwad SK. Microglia dictate the impact of saturated fat consumption on hypothalamic inflammation and neuronal function. Cell Rep. (2014) 9:2124–39. 10.1016/j.celrep.2014.11.018 25497089 PMC4617309

[21] Valdearcos M Douglass JD Robblee MM Dorfman MD Stifler DR Bennett ML. Microglial inflammatory signaling orchestrates the hypothalamic immune response to dietary excess and mediates obesity susceptibility. Cell Metab. (2017) 26:185–97.e3. 10.1016/j.cmet.2017.05.015 28683286 PMC5569901

[22] Hotchkiss RS Karl IE. The pathophysiology and treatment of sepsis. N Engl J Med. (2003) 348:138–50. 10.1056/NEJMra021333 12519925

[23] Stranberg L Verdrengh M Enge M Andersson N Amu S Önnheim K Mice chronically fed high-fat diet have increased mortality and disturbed immune response in sepsis. PLoS ONE. (2009) 4:e7605 10.1371/journal.pone.0007605 19865485 PMC2765728

[24] Huang H Liu T Rose JL Stevens RL Hoyt DG. Sensitivity of mice to lipopolysaccharide is increased by a high saturated fat and cholesterol diet. J Inflamm. (2007) 4:22. 10.1186/1476-9255-4-22 17997851 PMC2186306

[25] Sakai S Iizuka N Fujiwara M Miyoshi M Aoyama M Maeshige N. Mild obesity reduces survival and adiponectin sensitivity in endotoxemic rats. J Surg Res. (2013) 185:353–63. 10.1016/j.jss.2013.06.002 23838384

[26] Lawrence CB Brough D Knight EM. Obese mice exhibit an altered behavioural and inflammatory response to lipopolysaccharide. Dis Model Mech. (2012) 5:649–59. 10.1242/dmm.009068 22328591 PMC3424462

[27] DeMartini T Nowell M James J Williamson L Lahni P Shen H. High fat diet-induced obesity increases myocardial injury and alters cardiac STAT3 signaling in mice after polymicrobial sepsis. Biochim Biophys Acta Mol Basis Dis. (2017) 1863:2654–60. 10.1016/j.bbadis.2017.06.008 28625915 PMC5653424

[28] Andersson U Tracey KJ. Reflex principles of immunological homeostasis. Annu Rev Immunol. (2012) 30:313–35. 10.1146/annurev-immunol-020711-075015 22224768 PMC4533843

[29] Liu TF Vachharajani VT Yoza BK McCall CE. NAD+-dependent sirtuin 1 and 6 proteins coordinate a switch from glucose to fatty acid oxidation during the acute inflammatory response. J Biol Chem. (2012) 287:25758–69. 10.1074/jbc.M112.362343 22700961 PMC3406663

[30] Liu TF Yoza BK El Gazzar M Vachharajani VT McCall CE. NAD+-dependent SIRT1 deacetylase participates in epigenetic reprogramming during endotoxin tolerance. J Biol Chem. (2011) 286:9856–64. 10.1074/jbc.M110.196790 21245135 PMC3058977

[31] Vachharajani VT Liu TF Wang X Hoth JJ Yoza BK McCall CE. Sirtuins link inflammation and metabolism. J Immunol Res. (2016) 2016:8167273. 10.1155/2016/8167273 26904696 PMC4745579

[32] Vachharajani VT Russell JM Scott KL Conrad S Stokes KY Tallam L. Obesity exacerbates sepsis-induced inflammation and microvascular dysfunction in mouse brain. Microcirculation. (2005) 12:183–94. 10.1080/10739680590904982 15828130

[33] Borovikova LV Ivanova S Zhang M Yang H Botchkina GI Watkins LR. Vagus nerve stimulation attenuates the systemic inflammatory response to endotoxin. Nature. (2000) 405:458–62. 10.1038/35013070 10839541

[34] Gallowitsch-Puerta M Tracey KJ. Immunologic role of the cholinergic anti-inflammatory pathway and the nicotinic acetylcholine alpha 7 receptor. Ann N Y Acad Sci. (2005) 1062:209–19. 10.1196/annals.1358.024 16461803

[35] Wang D Zhou R Yao Y. Role of cholinergic anti-inflammatory pathway in regulating host response and its interventional strategy for inflammatory diseases. Chin J Traumatol. (2009) 12:355–64. 10.3760/cma.j.issn.1008-1275.2009.06.007 19930906

[36] Van Westerloo DJ. The vagal immune reflex: a blessing from above. Wiener Medizinische Wochenschrift. (2010) 160:112–7. 10.1007/s10354-010-0761-x 20364413

[37] Peña G Cai B Liu J van der Zanden EP Deitch E a de Jonge WJ. Unphosphorylated STAT3 modulates alpha 7 nicotinic receptor signaling and cytokine production in sepsis. Eur J Immunol. (2010) 40:2580–9. 10.1002/eji.201040540 20706987 PMC3086065

[38] St-Pierre S Jiang W Roy P Champigny C LeBlanc É Morley BJ. Nicotinic acetylcholine receptors modulate bone marrow-derived pro-inflammatory monocyte production and survival. PLoS ONE. (2016) 11:1–18. 10.1371/journal.pone.0150230 26925951 PMC4771711

[39] Wang L Opland D Tsai S Luk CT Schroer SA Allison MB. Pten deletion in RIP-cre neurons protects against type 2 diabetes by activating the anti-inflammatory reflex. Nat Med. (2014) 20:484–92. 10.1038/nm.3527 24747746

[40] Aomatsu M Kato T Kasahara E Kitagawa S. Gender difference in tumor necrosis factor-α production in human neutrophils stimulated by lipopolysaccharide and interferon-γ. Biochem Biophys Res Commun. (2013) 441: 220–5. 10.1016/j.bbrc.2013.10.042 24140406

[41] Marriott I Bost KL Huet-Hudson YM. Sexual dimorphism in expression of receptors for bacterial lipopolysaccharides in murine macrophages: a possible mechanism for gender-based differences in endotoxic shock susceptibility. J Reprod Immunol. (2006) 71:12–27. 10.1016/j.jri.2006.01.004 16574244

[42] Rettew JA Huet-Hudson YM Marriott I. Testosterone reduces macrophage expression in the mouse of toll-like receptor 4, a trigger for inflammation and innate immunity. Biol Reprod.(2008) 78:432–37. 10.1095/biolreprod.107.063545 18003947

[43] Klein SL Flanagan KL. Sex differences in immune responses. Nat Rev Immunol. (2016) 16:626–38. 10.1038/nri.2016.90 27546235

