# Supplementary figures and images for: Short-Term High-Fat Diet Consumption Reduces Hypothalamic Expression of the Nicotinic Acetylcholine Receptor α7 Subunit (α7nAChR) and Affects the Anti-inflammatory Response in a Mouse Model of Sepsis
Source: Front Immunol. 2019 Mar 22;10:565. doi: 10.3389/fimmu.2019.00565 (PMC6438922; doi:10.3389/fimmu.2019.00565)

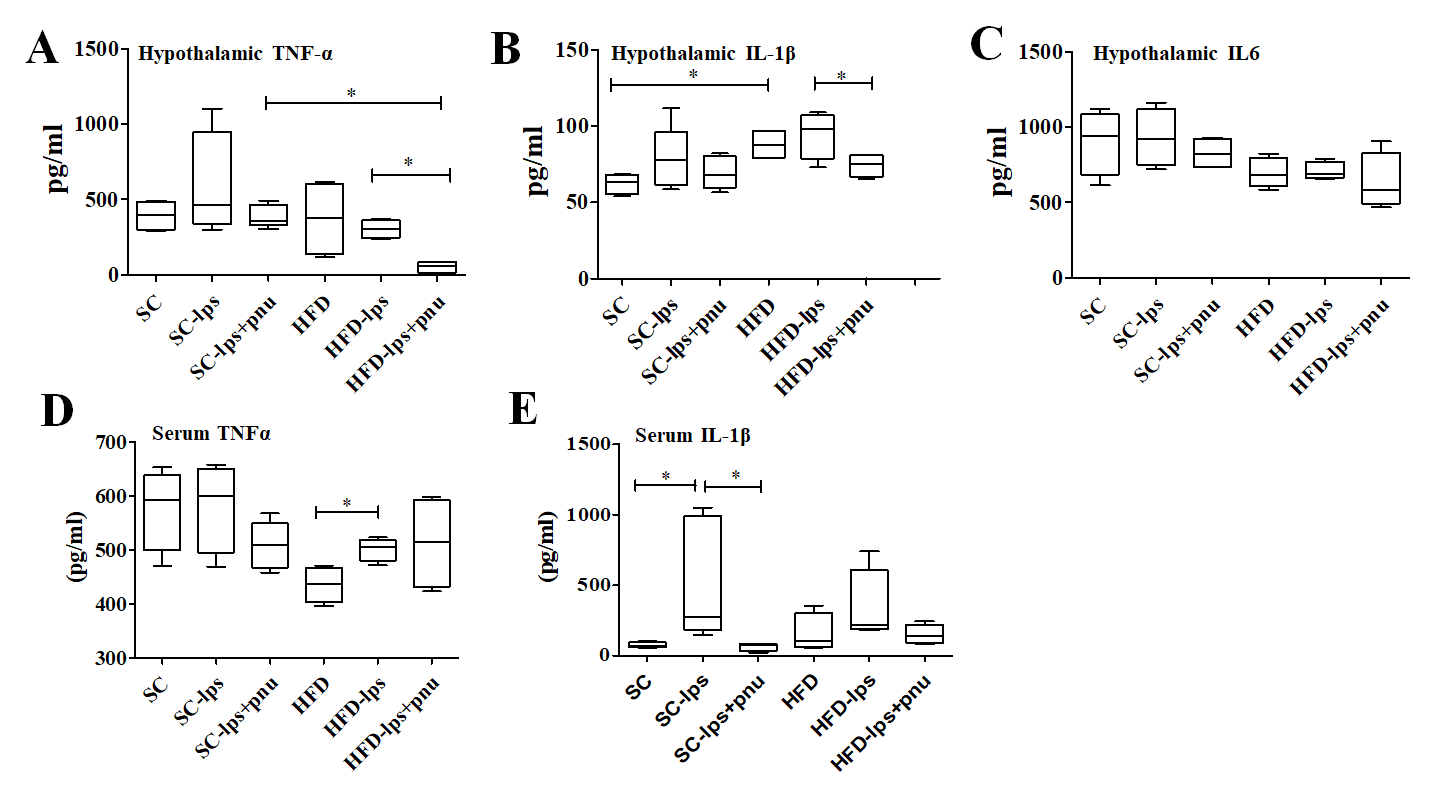

Supplement: Figure S1 — Hypothalamic (A–C) and serum cytokine levels (D,E). Quantitative Elisa (A), TNF-α, (B), Il-1β and (C). IL6 levels in hypothalamus and (D), TNF-α and (E). Il-1β levels in serum of mice injected intraperitoneally with a high dose of LPS (12 mg/kg) followed by stimulation intracerebroventricularly with PNU-282987 (10 pmoles/mouse). Significant differences between individual groups are shown (ANOVA with post-hoc Tukey HSD test: *p < 0.05). [file Image_1.TIFF]

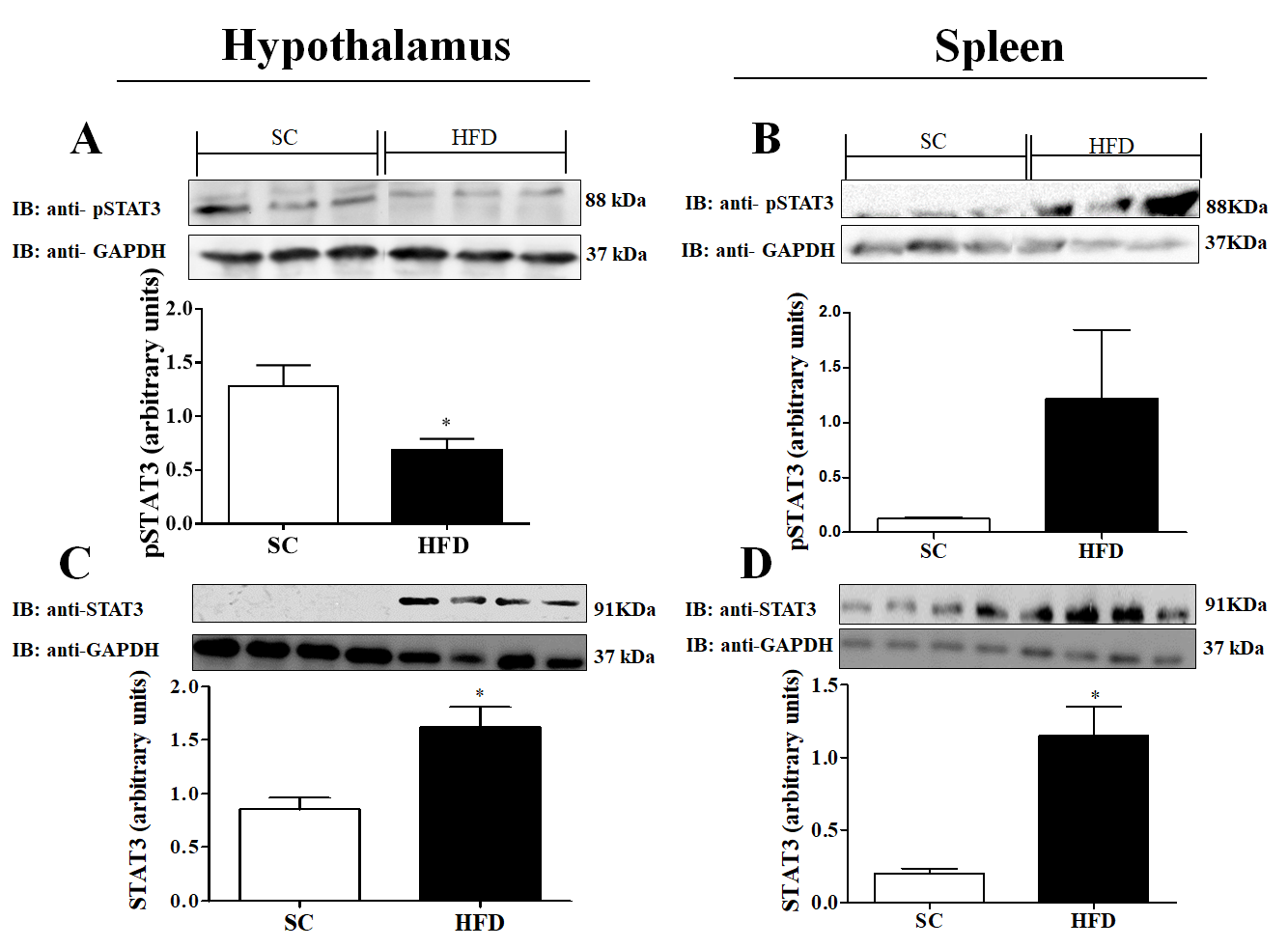

Supplement: Figure S2 — Protein content of pSTAT3 and STAT3 in the hypothalamus (A,C) and spleen (B,D) STAT3. Mice were fed standard chow diet (SC) or high fat diet (HFD) for 3 days. The bars represent the mean S.E.M. n = 3–4. *Means significantly different by unpaired t-tests (*p < 0.05). [file Image_2.TIFF]

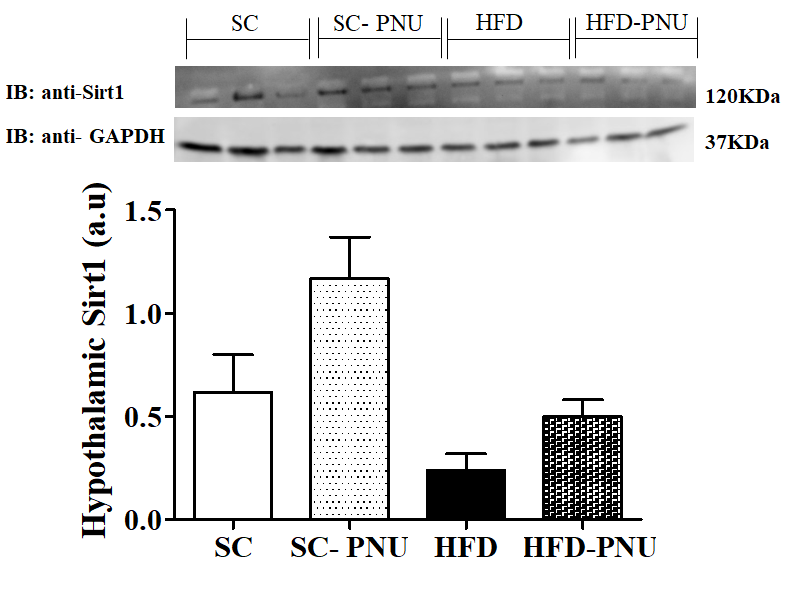

Supplement: Figure S3 — Hypothalamic SIRT1 level. Protein content of Sirt1 (SC, n = 3 and SC-PNU, n = 3; HFD, n = 3 and HFD-PNU, n = 3) in the hypothalamus of mice fed standard chow diet (SC) or high fat diet (HFD) for 3 days and/or received icv injection of the PNU (10 pmoles/mouse). The bars represent the mean S.E.M. *Means significantly different by ANOVA with post-hoc Tukey HSD test: *p < 0.05. [file Image_3.TIFF]
